# Quality-of-Service-Aware and Energy-Efficient Cluster-Based Routing Protocol for Reliable Data Transfer in the Internet of Underwater Things

**DOI:** 10.3390/s26217058

**Published:** 2026-11-08

**Authors:** Sabeen Arain, Sarang Karim, Intesab Hussain Sadhayo, Sherali Zeadally, Lubna Luxmi Dhirani

**Affiliations:** 1 Department of Information Technology, Quaid-e-Awam University of Engineering, Science and Technology, Nawabshah 67450, Pakistan; asabeen17@gmail.com; 2 Faculty of Pharmaceutical Sciences, People’s University of Medical and Health Sciences for Women (PUMHSW), Nawabshah 67450, Pakistan; 3 Department of Electronic and Computer Engineering, University of Limerick, V94 T9PX Limerick, Ireland; lubna.luxmi@ul.ie; 4 Department of Computer Systems Engineering, Quaid-e-Awam University of Engineering, Science and Technology, Nawabshah 67450, Pakistan; intesab@quest.edu.pk; 5 College of Communication and Information, University of Kentucky, Lexington, KY 40506, USA; szeadally@uky.edu; 6 Academy of Computer Science & Software Engineering, University of Johannesburg, Johannesburg 2006, South Africa

**Keywords:** cluster-based routing, energy-efficient routing, hierarchical routing, multi-criteria decision-making (MCDM), QoS-aware routing, internet of underwater things (IoUT)

## Abstract

The Internet of Underwater Things (IoUT) supports marine applications such as environmental monitoring, offshore exploration, disaster prevention, and underwater infrastructure inspection. However, limited acoustic bandwidth, long propagation delays, unreliable communication links, and constrained node energy make reliable and energy-efficient routing challenging. We propose a Quality-of-Service (QoS)-Aware and Energy-Efficient Cluster-Based Routing (QEE-CBR) protocol that integrates cylindrical three-dimensional network partitioning, multi-criteria cluster head selection, hierarchical forwarding, and in-network data aggregation. The proposed Multi-Criteria Decision-Making (MCDM) mechanism selects cluster heads using residual energy, node degree, node proximity, and centroid distance, while regional cluster heads coordinate upward data forwarding toward a single surface sink. We implemented QEE-CBR in Network Simulator 3 (NS-3). We evaluated it against four benchmark protocols: Balanced Energy-Efficient Clustering (BEEC), Multi-Level Clustering Energy-Efficient Routing (MLCEE), Reliable QoS-Aware Routing (RQAR), and Energy-Aware Void Avoidance Routing Protocol (EAVARP). The performance metrics used included packet delivery ratio, throughput, end-to-end delay, network lifetime, and energy consumption for varying network densities. We conducted additional experiments to investigate the contributions of individual protocol components through ablation analysis, assess the sensitivity of routing performance to selected parameters, and compare the proposed clustered hierarchical architecture with our previous QoS-Aware and Energy-Optimised Cooperative Opportunistic Routing Protocol (QE-ORP). At 500 nodes, QEE-CBR achieves a packet delivery ratio of 99%, a throughput of 425 kbps, an end-to-end delay of 0.15 s, and a first-node-death time of 1130 s, along with a total energy consumption of 730 J. The architectural comparison highlights the distinction between QE-ORP’s flat cooperative opportunistic forwarding and QEE-CBR’s spatially organised hierarchical routing. The results obtained provide a comparative simulation-based assessment of the proposed protocol, its cluster head selection mechanism, and the effects of its principal design choices under the configurations used in our evaluation.

## 1. Introduction

The Internet of Underwater Things (IoUT) has emerged as an enabling technology for a wide range of marine applications, including environmental monitoring, offshore oil and gas exploration, disaster prevention, military surveillance, underwater infrastructure inspection, and oceanographic data collection [1,2]. The IoUT supports distributed sensing and underwater communication, which enable continuous monitoring of marine environments that are hard to monitor with conventional communication technologies. This has made the IoUT an important research area with respect to the development of smart and sustainable marine monitoring systems. Radio-frequency signals are highly attenuated in water, whereas optical signals are scattered and absorbed over long distances, so underwater communication is mainly based on acoustic signals [3]. The features of acoustic communication are limited bandwidth, long propagation delays, high bit error rates, multipath fading, and Doppler effects [4,5]. In addition, underwater sensor nodes have a limited battery size, and replacement or recharging of the batteries is often not feasible after deployment. These attributes make reliable and energy-efficient routing significantly more difficult in IoUT networks compared to terrestrial networks [6].

Current IoUT routing protocols aim to enhance the reliability of communication, energy efficiency, network lifetime, or scalability [7,8]. Geographic and opportunistic routing protocols utilize geographic information, such as node location, depth, or forwarding probability, to make forwarding decisions [9]. These approaches provide a solid foundation and can be further bolstered by cluster-based organisation that minimises redundant transmissions, balances the communication load, and increases scalability in dense deployments. Thus, cluster-based routing has gained much attention because it provides hierarchical grouping of nodes so that cluster heads can manage intra-cluster and inter-cluster communication, minimising intra-cluster and inter-cluster communication overheads, as well as energy consumption, and maximising network lifetime [10,11]. The energy consumption, delay, and reliability of communication are influenced by the multi-hop IoUT framework and hierarchical clustering, where the burden of forwarding is distributed more evenly between the clusters, and by the cluster size, the location of the cluster head, and the distance between cluster heads and the sink. Thus, an effective cluster-based architecture should consider not only the energy status of the candidate cluster heads but also the connectivity and spatial attributes of the cluster heads.

In this work, we build upon these ideas and propose a new protocol for IoUT: Quality-of-Service (QoS)-Aware and Energy-Efficient Cluster-Based Routing (QEE-CBR). The protocol uses a hierarchical cylindrical network partitioning mechanism and a Multi-Criteria Decision-Making (MCDM)-based cluster head selection strategy. Candidate cluster heads are evaluated using residual energy, node degree, node proximity, and centroid distance to support balanced energy utilisation and stable cluster formation. Multi-level hierarchical forwarding and data aggregation are also incorporated to reduce communication overhead and support scalable data delivery toward the surface sink. We developed our proposed protocol and simulated it in Network Simulator 3 (NS-3). We evaluated the protocol against the four benchmark IoUT routing protocols, including Balanced Energy-Efficient Clustering (BEEC) [12], Multi-Level Clustering Energy-Efficient Routing (MLCEE) [13], Reliable QoS-Aware Routing (RQAR) [14], and Energy-Aware Void Avoidance Routing Protocol (EAVARP) [15].

### 1.1. Motivation for Cylindrical Geometry

The three-dimensional deployment of underwater sensor nodes requires a spatial partitioning strategy that must express both the depth and the radial position from the surface sink. For vertical anchor deployments or for radial sensor deployments or layered underwater deployments, the natural network structure is a cylindrical partitioning of the application [16]. It arranges the sensors by depth and radial distance from a central reference point, facilitates cluster formation, and simplifies the identification of cluster heads in the correct place. Three-dimensional deployments can also be spherical [12] or cuboidal [17], and each has different cluster shapes and inter-cluster relationships. Cylindrical segmentation is adopted in QEE-CBR as a suitable geometric basis for the underwater deployment considered and for supporting hierarchical routing [18].

### 1.2. Research Gap and Contributions

#### 1.2.1. Research Gaps

Based on the reviewed literature in Section 2, we identified three research directions that motivated the development of QEE-CBR.

Research Direction 1 (RD1): Joint QoS and Energy Optimisation.

*Research Problem:* Existing routing protocols often prioritise a particular objective, such as energy efficiency or communication reliability, rather than jointly considering multiple QoS and energy requirements within a clustered and hierarchical routing architecture [19].

*Research Opportunity:* Integrating packet delivery ratio, throughput, end-to-end delay, network lifetime, and energy consumption into a unified routing design provides an opportunity to balance communication performance and energy efficiency. In our previous work [20], we developed the QoS-Aware and Energy-Optimised Cooperative Opportunistic Routing Protocol (QE-ORP), which selects relay nodes using depth, residual energy, link quality, and packet delivery probability. Building on these relay-selection and multi-objective optimisation principles, we investigate their extension within a hierarchical, cluster-based routing framework to improve energy efficiency and communication reliability in underwater wireless sensor networks.

Research Direction 2 (RD2): Multi-Criteria Cluster Head Selection.

*Research Problem:* When cluster heads are selected based on residual energy alone, we do not consider the topology, connectivity, and spatial relations between nodes [21].

*Research Opportunity:* We select cluster heads based on multiple criteria, including residual energy, node degree, node proximity, and centroid distance, thereby improving the selection of cluster heads. Incorporating energy information along with the topological information can help with the appropriate placement of cluster heads, energy balance, and stable cluster formation.

Research Direction 3 (RD3): Integration of Hierarchical Routing and Spatial Organisation.

*Research Problem:* Clustering, hierarchical forwarding, energy efficiency, communication reliability, and scalability are generally not integrated into one routing architecture. Some efforts are focused on the concept of a local cluster that is not hierarchical but, rather, has clusters of nodes that are clustered together [14], while others are based on predefined or static deployment structures [13].

*Research Opportunity:* A structured method for underwater data collection and forwarding toward a single surface sink is to incorporate cylindrical network partitioning into levels, tracks, sectors, and blocks, along with local clustering, regional cluster heads, hierarchical upward forwarding, and in-network data aggregation. It is worth noting here that the research opportunity is not about new mechanisms but, rather, the integration of these mechanisms into a single routing architecture.

#### 1.2.2. Contributions of This Work

This study makes the following four research contributions to solve the research problems identified above:

- Contribution 1 (addresses RD1): We design a new routing protocol, called QEE-CBR, that incorporates QoS requirements and energy constraints in a clustered, hierarchical forwarding structure. It takes into account packet delivery ratio, throughput, end-to-end delay, network lifetime, and energy consumption, and it extends the design perspective of our previous QE-ORP protocol from flat cooperative opportunistic forwarding to structured hierarchical cluster-based routing.
- Contribution 2 (addresses RD2): We propose an MCDM-based cluster head selection method, which considers the residual energy, node degree, node proximity, and centroid distance. This mechanism includes both the energy and the topological information in the selection process, aiming to balance energy consumption and a stable cluster formation.
- Contribution 3 (addresses RD3): We develop a strategy of partitioning the three-dimensional deployment in order to organise the deployment into levels, tracks, sectors, and blocks. This approach, when combined with local cluster formation, regional cluster heads, hierarchical upward forwarding, and in-network data aggregation, provides an integrated approach for the collection and forwarding of structured data toward a single surface sink. As we mentioned before, the contribution here is the integration of these mechanisms in QEE-CBR.
- Contribution 4 (evaluates RD1–RD3): We implement QEE-CBR in the NS-3 simulator and evaluate its performance against four benchmark routing protocols under different network densities. We also conduct component ablation and parameter-sensitivity experiments to examine the effects of selected design choices on the performance metrics. These experiments provide a simulation-based evaluation of the proposed architecture and its principal components under the specified configuration.

We organise the remainder of this paper as follows: Section 2 reviews existing energy-efficient, QoS-aware, clustering, and hierarchical routing approaches for the IoUT. Section 3 presents the system model, problem formulation, network assumptions, and acoustic communication models. Section 4 describes the proposed protocol, including the cylindrical network partitioning, cluster head selection, and hierarchical routing mechanisms. Section 5 presents the simulation environment and configuration used for the evaluation. Section 6 presents the experimental results, the sensitivity and ablation analyses, and the application context of the proposed protocol. Section 7 discusses the results, acknowledges the limitations of the present study, and outlines future research directions. Finally, Section 8 concludes the paper.

## 2. Related Work

QoS-aware and energy-efficient routing remain important research directions in the IoUT because underwater networks operate under constrained bandwidth, relatively long propagation delays, unreliable acoustic links, and limited node energy. Existing routing protocols address energy consumption [5,9,19], communication reliability [22,23,24], routing voids [15,25,26], and network lifetime [11,16,27,28]. These routing protocols can be roughly grouped into the following categories: clustering-based routing, opportunistic routing, geographic routing, and hybrid routing [29,30,31]. Clustering-based protocols are especially relevant to dense deployments because they organise sensor nodes into groups, enable cluster heads to coordinate communication within the group, and aggregate data prior to sending it to the sink [27,32]. Their performance relies on the cluster formation, cluster head selection, and the organisation of inter-cluster forwarding, which jointly affect reliability and efficiency [21].

Recently, learning-based and cross-layer underwater routing methods have also been studied. For underwater acoustic networks, the author of [33] presented an Artificial Intelligence (AI)-based routing survey based on machine learning, reinforcement learning, and fuzzy logic methods, and other works studied adaptive underwater forwarding using Q-learning and multi-agent reinforcement learning methods [34,35]. Simultaneous study of routing reliability, load balancing, and energy efficiency has been proposed in the context of cross-layer approaches [36]. These studies explore the design space beyond the traditional rule-based routing and answer questions complementary to those explored in QEE-CBR. The goal of the present work is not to claim novelty in terms of learning but to assess whether the explicit cylindrical spatial organisation, the multi-criteria cluster head selection, the regional hierarchical forwarding, and aggregation offer measurable advantage under a common configuration during simulation.

BEEC [12] divides the network into circular sub-regions and uses two mobile sinks with clockwise movement to achieve better coverage and energy efficiency. The protocol enhances the network lifetime, stability period, energy usage, and throughput when compared to some of the static-sink approaches. In addition to the movement, synchronisation, and route management considerations of the mobile sink, there are some deployment scenarios where complementary static-sink designs are useful.

MLCEE [13] partitions underwater sensor nodes into layers, forms clusters within individual layers, and selects cluster heads using residual energy and Bayesian probability. Its hierarchical design aims to balance energy consumption and mitigate energy hotspots near the surface sink. However, the cluster head selection and inter-cluster forwarding mechanisms provide opportunities for further investigation. Specifically, incorporating more explicit assessments of communication quality, node connectivity, and spatial distribution into the Bayesian-probability-based cluster head selection mechanism could improve the suitability of selected cluster heads under varying network conditions. Furthermore, adaptive cluster formation and cluster-head reselection could be investigated to maintain routing efficiency as node positions, connectivity, and residual energy change over time.

RQAR [14] uses the MCDM strategy together with the Technique for Order Preference by Similarity to an Ideal Solution (TOPSIS) [37] to select the forwarding nodes based on link quality, hop count, congestion level and residual energy. RQAR improves routing performance by evaluating candidate forwarders against multiple quality-of-service and energy-related criteria. It also mitigates communication voids (regions in which a node cannot identify a suitable next-hop forwarder towards the sink) through adaptive transmission ranges and sender-side holding times. Although RQAR organises nodes into virtual layers according to their hop distance from the mobile sink, it does not employ an explicit hierarchical cluster-based routing structure. Extending this virtual-layer organisation with structured clusters and cluster head-based forwarding could therefore provide an opportunity to reduce redundant forwarding and control routing overhead.

EAVARP [15] addresses the flooding, cyclic forwarding, and routing voids by mixing the concentric layers with an Opportunistic Directional Forwarding Strategy (ODFS). The protocol considers residual energy and energy related to transmission in forwarding decisions. It enhances the reliability of communication when voids exist, and the multi-candidate forwarding design provides potential for further optimisation of redundant transmissions and energy use.

QoS-Aware Evolutionary Routing Protocol (QERP) [38] uses evolutionary clustering to minimise propagation delay, noise, and multipath effects. It outperforms the selected clustering approaches in terms of packet delivery ratio, energy usage, and end-to-end delay. Evolutionary optimisation provides a flexible search mechanism, and lightweight variants that are suitable for resource-constrained underwater nodes are an interesting path to further explore.

The Anchor-Nodes-Assisted Cluster-Based Routing Protocol (ANCRP) [17] partitions the deployment region into cubic clusters and uses anchor nodes to enhance the data forwarding reliability. A void-handling mechanism dynamically chooses temporary cluster heads to keep the networks connected. The protocol demonstrates how the benefit of geometric clustering and auxiliary forwarding nodes can be leveraged, and the reduction in the extra architectural elements can reduce the routing complexity.

Energy-Aware Multi-Level Clustering Scheme (EMLC) [16] uses a three-dimensional concentric cylindrical topology to organise nodes into multi-level clusters and employs vertical bottom–up communication to minimise energy loss close to the seabed. The remaining energy is used to select cluster heads. Both EMLC and the present study are related to each other with respect to their cylindrical organisation of space and hierarchical communication. QEE-CBR extends the cluster head selection process by considering residual energy, node degree, node proximity, and centroid distance together, giving different approaches different cluster head selection criteria and cluster organisation.

QoS-Based Void Avoidance Routing Technique (QoS-VAR) [39] mitigates communication voids by considering node depth, distance, holding time, and remaining energy when selecting a two-hop forwarding neighbour. The simulation-based results indicate that the protocol enhances the packet delivery ratio, energy efficiency, and node survivability. The design focuses on void avoidance, and multi-level organisation is a logical extension to further enhance structural scalability.

### Related Clustering and Architectural Comparators

The proposed QEE-CBR closely relates to the Floating-Nodes-Assisted Cluster-Based Routing (FNCBR) [40], which uses hierarchical data collection and geometric partitioning. The deployment area is subdivided into cuboidal sub-regions, with floating nodes placed on the surface of each region, and fixed cluster heads placed in different layers. In contrast, QEE-CBR uses cylindrical partitioning, a multiple-criteria local cluster head selection method, four fixed cluster heads at each level of the network spread throughout the region, acoustic forwarding between underwater nodes, and a single surface sink. FNCBR is considered an architectural comparator that illustrates an alternative geometric and hierarchical organisation.

Our previous work, QE-ORP [20], provides an important comparison because it also targets QoS, reliability, and energy efficiency in underwater wireless sensor networks. QE-ORP employs cooperative opportunistic forwarding, where eligible relay nodes are selected according to forwarding-related information such as node depth, residual energy, and link conditions. QE-ORP does not use explicit local clusters, fixed regional cluster heads, or the level–track–sector–block (L,T,S,B) spatial organisation employed by QEE-CBR, and its forwarding architecture is therefore fundamentally different from the hierarchical cluster-based organisation considered in this work.

QEE-CBR combines these architectural elements by first partitioning the cylindrical deployment into levels, tracks, sectors, and blocks, followed by local cluster head selection using residual energy, node degree, centroid proximity, and centroid distance. Data is aggregated at local cluster heads and subsequently forwarded through the regional cluster head hierarchy toward the single surface sink. The resulting architecture provides a structured alternative to both flat cooperative forwarding and conventional energy-based clustering. Table 1 summarises the main architectural and routing characteristics of the reviewed protocols and the proposed QEE-CBR protocol. The comparison focuses on the differences in clustering, cluster head selection, routing strategy, quality-of-service support, hierarchical organisation, geometric structure, and scalability, without claiming that the individual mechanisms incorporated into QEE-CBR are themselves novel.

## 3. Preliminaries

This section introduces the system model, problem formulation, network assumptions, acoustic communication models, and energy model used throughout the proposed protocol. To maintain mathematical consistency, we define all symbols and variables once and use them consistently in subsequent sections.

### 3.1. Problem Formulation

Underwater acoustic communication has several key features that make the problem of reliable underwater routing substantially more challenging than the terrestrial wireless sensor network case. Bandwidth, propagation delays, attenuation, multipath fading, Doppler spread, and high transmission energy consumption are issues associated with acoustic channels. Electrical power is also a problem for underwater sensor nodes because batteries are usually difficult or impossible to recharge after the nodes are deployed. Therefore, routing protocols should reduce communication overhead, ensure reliable packet delivery, and extend the network lifetime.

To overcome these problems, we propose a hierarchical cluster-based routing protocol based on cylindrical network partitioning along with cluster head selection using MCDM. The proposed routing strategy considers the reliability of communication, energy consumed, scalability of the network, and packet forwarding efficiency.

### 3.2. System Model

**Figure 1:**
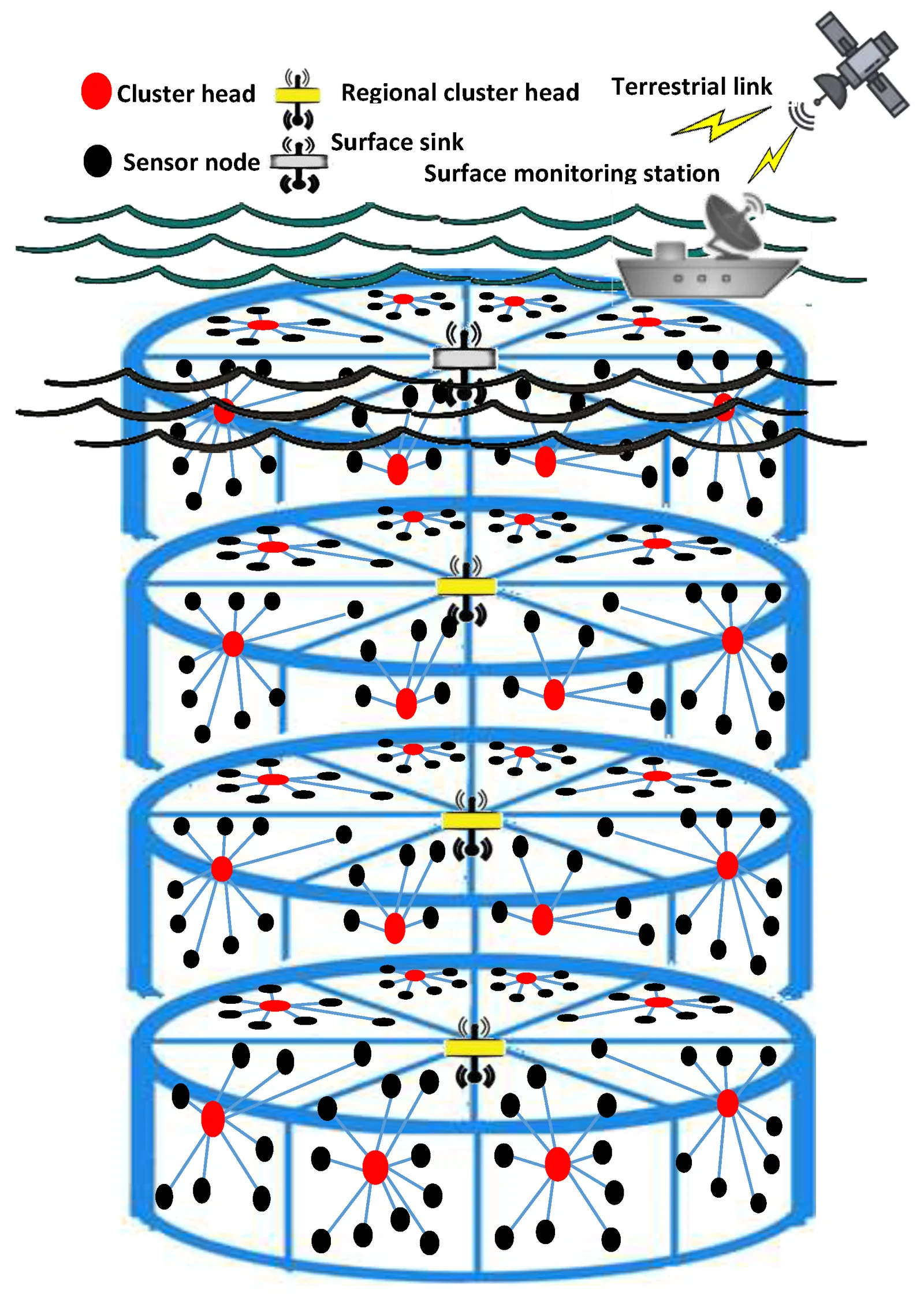
3D cylindrical network architecture for the proposed protocol, which shows the surface monitoring station, the single surface sink at the cylinder axis, four fixed regional cluster heads (one per layer), and the distributed underwater sensor nodes.

Our proposed routing protocol considers a 3D underwater monitoring region represented by a right circular cylinder of radius *R* and height *H*, as shown in Figure 1. The deployment consists of four major components [16,20]: a surface monitoring station, a surface sink, regional cluster heads, and sensor nodes. All underwater communication is performed using acoustic links, whereas radio-frequency communication is employed only between the surface sink and the monitoring station.

#### 3.2.1. Surface Monitoring Station

The surface monitoring station is the final destination for all sensed information. It receives aggregated data from the surface sink through high-speed radio-frequency communication for storage, visualisation, and further analysis.

#### 3.2.2. Surface Sink

A surface sink is positioned at the water surface and serves as the interface between the underwater acoustic network and the terrestrial monitoring infrastructure. It receives aggregated packets from the regional cluster heads through acoustic communication and forwards them to the monitoring station using radio communication.

#### 3.2.3. Regional Cluster Heads

Regional cluster heads are deployed at the centre of each depth level. Their primary responsibility is to aggregate data received from local cluster heads and relay the aggregated packets toward the surface sink through hierarchical multi-hop communication. Since regional cluster heads participate in inter-layer communication, they reduce long-distance acoustic transmissions and improve network scalability. Only one regional cluster head is placed at the centre depth of each of the four vertical levels, giving four fixed regional cluster heads in total.

#### 3.2.4. Cluster Heads

For the upper layer, sensor nodes pass data to cluster heads, which aggregate the data and pass it to the upper layer. They are chosen from sensor nodes according to several criteria, which are residual energy, node degree, proximity to the neighbouring nodes, and centroid distance [21]. Cluster heads are chosen as the most suitable nodes that are capable of collecting and forwarding data. The most appropriate nodes are those in the cluster that satisfy the following properties: they have high residual energy, high node degree, and central positions in the cluster, which is why these nodes are most suitable for data aggregation and reliable forwarding [17]. The selection is made by the decision function, which is based on the concept of MCDM and is defined in Equation (22).

#### 3.2.5. Sensor Nodes

Sensors are randomly distributed in each of the blocks using cylindrical coordinates. The nodes are source nodes under the proposed routing protocol, and they continuously check the underwater environment, sense the physical parameters, and generate the data packets to send out. The sensed data is then transferred to the cluster heads by acoustic communication links. All nodes in the network are assumed to have identical hardware and communication capabilities, including a unique identifier, the same initial energy level, and the same communication range.

### 3.3. Assumptions

Based on the IoUT literature [16,41], we make the following assumptions:

- Sensor nodes (totalling between 100 and 500) are deployed randomly within a right circular cylinder of radius 500 m and height 1000 m, embedded in a 1000×1000×1000 m^3^ bounding box.
- The cylinder is divided into four vertical levels, one radial track, eight sectors, and four radial blocks per sector. The spatial identifier is (L,T,S,B); the node identifier is a separate unique identifier.
- One fixed regional cluster head is placed at the centre of each level, giving four regional cluster heads in total. A single surface sink is positioned at the cylinder axis at the water surface.
- All underwater communication uses acoustic links. The sink and monitoring station use the radio communication link to communicate; no additional underwater gateway layer is introduced.
- Sensor nodes follow the stated random-walk mobility model, whereas the regional cluster heads and surface sink remain fixed.
- Node coordinates are estimated using a Time-of-Arrival (ToA)-based localisation procedure [42]. A separate Gaussian localisation-noise experiment uses σ=10 m.
- The acoustic transmission range, queue size, retry policy, packet generation rate, cluster head rotation threshold, and random-seed policy are fixed.

### 3.4. Acoustic Models

Acoustic signalling serves as the communication mechanism between underwater nodes. The formulation explicitly separates acoustic propagation loss from electrical energy-state quantities so that acoustic source levels expressed in dB re 1μPa are not interpreted as electrical watts.

#### 3.4.1. Thorp Propagation Model

The Thorp propagation model [43,44] is used to represent distance- and frequency-dependent acoustic attenuation. For a link of distance *d* metres, $d_{\mathrm{km}}=d/1000$ is used in the absorption term. The transmission loss relative to a 1 m reference distance is

(1) $$TL\left(d,f\right)=10\Omega \log_{10}\left(\frac{d}{d_{0}}\right)+\alpha \left(f\right)d_{\mathrm{km}},\;d_{0}=1\;m,$$

where Ω=1.5 is the practical spreading factor and α(f) is the Thorp absorption coefficient in dB/km. For *f* in kHz,

(2) $$\alpha \left(f\right)=\left\{\begin{array}{cc} \frac{0.11f^{2}}{1+f^{2}}+\frac{44f^{2}}{4100+f}+2.75\times 10^{-4}f^{2}+0.003, & f\geq 0.4,\\ 0.002+0.11\left(\frac{f}{1+f}\right)+0.011f, & f<0.4. \end{array}\right.$$

The corresponding linear loss factor is

(3) $$L\left(d,f\right)=10^{TL(d,f)/10}.$$

The absorption coefficients follow the widely used Thorp formulation [44,45]. The practical spreading factor, Ω=1.5, is adopted from the value reported in [46].

#### 3.4.2. Path Loss and Power Model

The propagation and energy models represent two related but distinct aspects of an underwater acoustic link. The Thorp model [44] determines the distance-dependent and frequency-dependent acoustic transmission loss, whereas the electrical energy model determines the energy consumed by the transmitter and receiver during each actual transmission or reception attempt. The two models are combined through the same link distance and through the packet-reception outcome; the acoustic loss is not converted directly into an electrical watt value.

For a candidate link of distance *d*, Equation (1) specifies the propagation loss. The received signal-to-noise ratio (SNR) is modelled as

(4) $$\mathrm{SNR}\left(d,f\right)=\frac{P_{tx}\;G}{N(f)\;L(d,f)},$$

where $P_{tx}$ is the transmit power, *G* is a lumped transmitter/receiver gain factor, N(f) is the ambient noise power spectral density at frequency *f*, and L(d,f) is the linear propagation loss from Equation (3). A packet is considered to be successfully received in the absence of collision when $\mathrm{SNR}\left(d,f\right)\geq \gamma_{0}$, where $\gamma_{0}$ is the receiver detection threshold. The ambient noise term N(f) follows the Wenz shipping, wind, and thermal noise model [47].

The distance-dependent electrical transmitter–amplifier power used by the energy model is defined separately from the acoustic propagation model as follows:

(5) $$P_{amp}\left(d\right)=E_{amp}\;R_{b}{\left(\frac{d}{d_{0}}\right)}^{\Omega },\;d_{0}=1\;m,$$

where $E_{amp}$ is the amplifier energy per bit at the reference distance, and $R_{b}$ is the nominal bit rate. This electrical amplifier term provides the distance dependence used in Equation (7); the Thorp loss in Equation (1) is used only for acoustic reception/SNR and is not added again as an electrical energy term.

#### 3.4.3. Energy Model

For a packet of *l* bits and nominal link data rate $R_{b}$, the modelled transmission and reception durations are

(6) $$T_{tx}=T_{rx}=\frac{l}{R_{b}}.$$

The per-attempt electrical energy is based on the underwater acoustic energy formulation [48,49,50]:

(7) $$\begin{array}{cc} E_{tx}\left(l,d\right) & =E_{elec}l+T_{tx}P_{amp}\left(d\right), \end{array}$$

(8) $$\begin{array}{cc} E_{rx}\left(l\right) & =E_{elec}l+T_{rx}P_{rx}, \end{array}$$

(9) $$\begin{array}{cc} E_{DA}\left(l\right) & =E_{DS}l. \end{array}$$

Here, $E_{elec}$ is the electronic energy coefficient, $P_{amp}\left(d\right)$ is the distance-dependent electrical transmitter–amplifier term defined in Equation (5), $P_{rx}$ is the receiver power term, and $E_{DS}$ is the data-processing/aggregation energy coefficient. These quantities are electrical energy-model parameters and should not be confused with the acoustic source level or the host-computer processing power. The electrical energy model follows the standard first-order radio formulation for wireless sensor networks [51]. The cumulative communication energy includes all actual transmission and reception attempts, including retransmissions. The residual energy of node *i* is therefore

(10) $$RE_{i}=E_{I}-\sum_{a\in A_{i}}E_{a},$$

where $E_{I}=5$ J denotes the initial energy assigned to each sensor node, and $A_{i}$ represents the transmission, reception, and aggregation operations performed by node *i*, each consuming energy $E_{a}$. Thus, a 500-node network has a total initial sensor-node energy of 2500 J under this assumption. The aggregate energy consumption of 730 J corresponds to the measurement interval considered in the evaluation. The regional cluster heads are assumed to be externally powered, and their energy consumption is not included in the sensor-node aggregate energy metric.

## 4. Proposed Routing Protocol

Figure 1 presents the architecture of the proposed routing protocol. When the network activates, all nodes are initially disconnected, and they are initialised by exchanging beacon messages. The optimal cluster heads for each cluster are selected using the MCDM method [14]. Through the beaconing process, information about the local reachability of the surface sink and the cluster heads is disseminated to the neighbouring cluster heads and the sensor nodes. Sensor nodes discover reachability information toward their corresponding cluster head within the block in which they reside, and neighbouring cluster heads obtain reachability information toward the surface sink. The protocol updates cluster heads regularly to ensure balanced resource utilisation and adapt to changing network conditions. After the beacon exchange, the sensor nodes send the collected data to the local cluster head. If a cluster head is out of the transmission range of a sink, the packets are forwarded through another cluster head on the way to the surface. For reliable intra-cluster and inter-cluster forwarding, we compare each candidate node’s fitness value, computed using the weighted MCDM decision function $F(V,CH_{i})$ defined in Equation (22), against a predefined QoS threshold. This threshold is determined empirically during simulation and is set to 0.5 based on initial calibration runs, balancing reliability and energy efficiency. Only nodes with a fitness value exceeding this threshold are considered eligible for packet forwarding. At the same time, the cluster heads aggregate the collected data to minimise energy consumption and overhead and maximise the network lifetime.

### 4.1. Protocol Operation Flow

**Figure 2:**
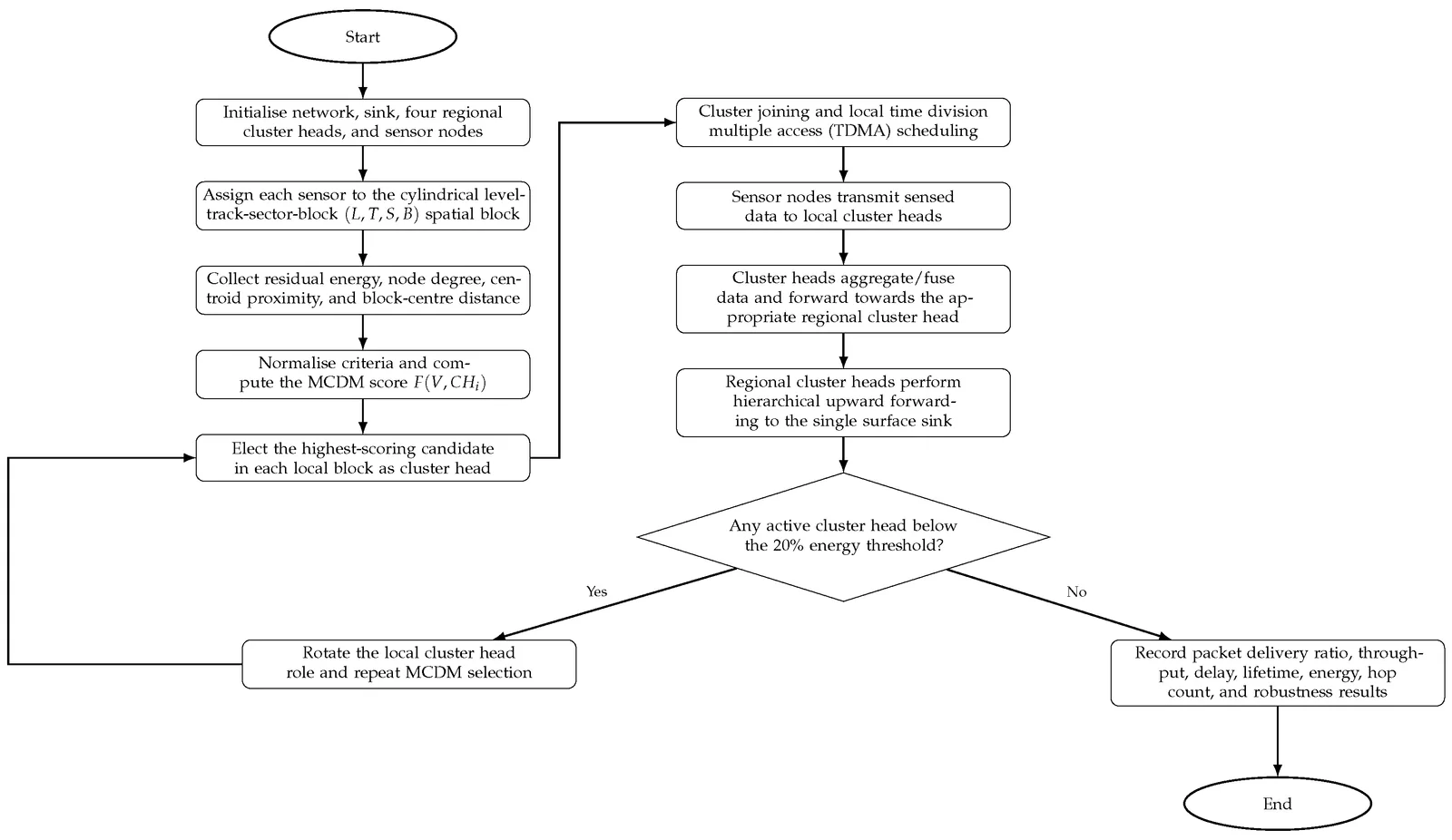
Operational flow of QEE-CBR from spatial deployment and MCDM-based cluster head selection to hierarchical data delivery and performance measurement.

Figure 2 shows the complete QEE-CBR operation, from deployment and spatial assignment to cluster head election, local aggregation, hierarchical forwarding, and performance measurement. The flowchart also shows the cluster head rotation condition and the single-sink termination point. This overview complements the detailed algorithms that we describe below.

### 4.2. Cylindrical Network Partitioning Scheme

The Cylindrical Network Partitioning Scheme (CNPS) provides a way of organising the underwater cylinder based on four spatial indices, namely, level (*L*), track (*T*), sector (*S*), and block (*B*). In this case, *L* stands for vertical level, *T* for radial track, *S* for angular sector, and *B* for radial block in an angular sector. The evaluated topology features one radial track, eight sectors, four radial blocks per sector, and four vertical levels, resulting in a total of 4×1×8×4=128 spatial blocks.

**Figure 3:**
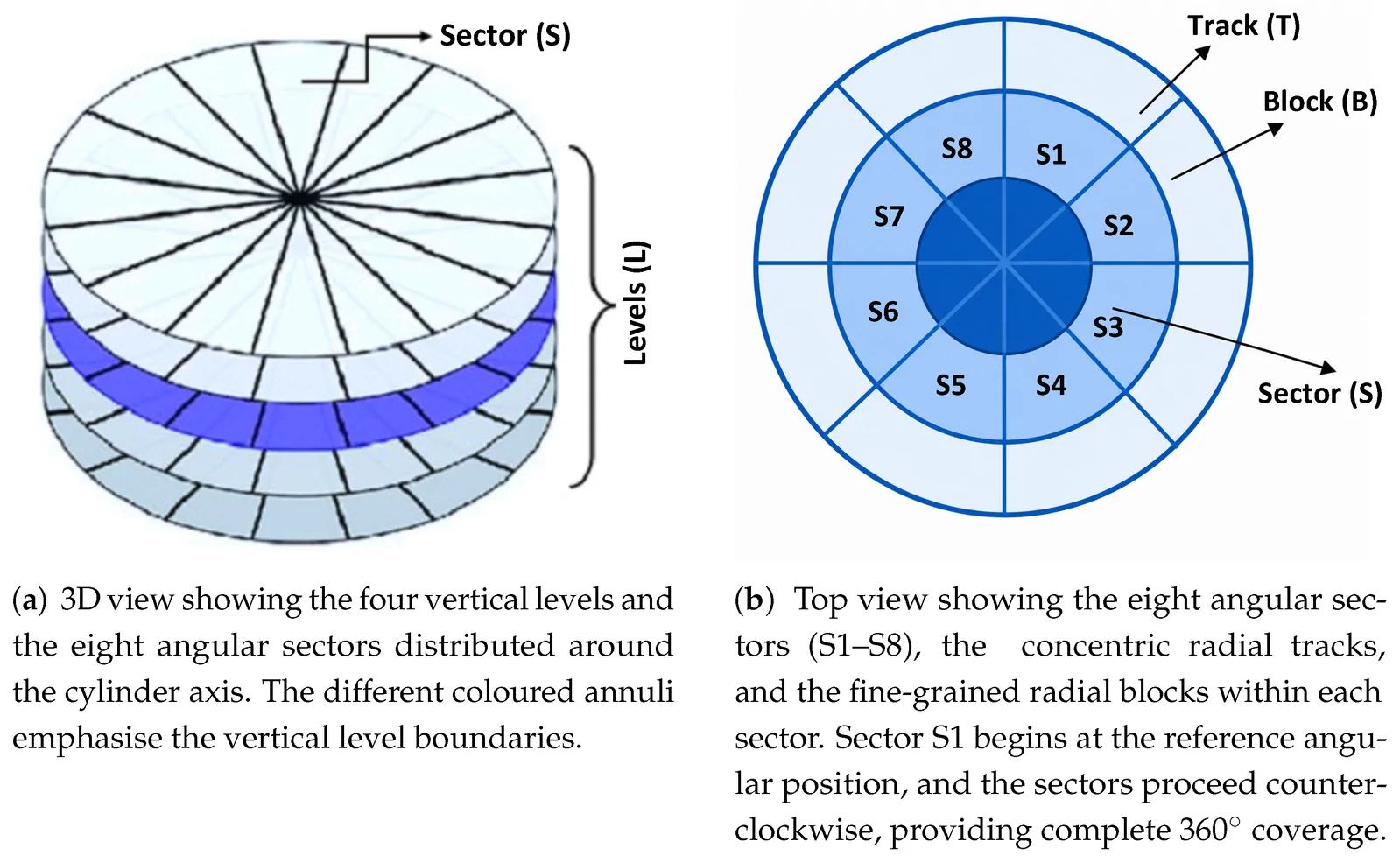
Cylindrical network partitioning into levels, tracks, sectors, and blocks. The 3D view in (**a**) and the top view in (**b**) together describe the full (L,T,S,B) spatial identifier used throughout the protocol. Sector labels S1–S8 correspond to the eight equal angular subdivisions described in Section 4.2.3.

Two complementary views of the same topology are provided in Figure 3. Figure 3a shows the 3D cylindrical volume, where the vertical axis is discretised into four level bands and the angular dimension is divided into eight sectors. Figure 3b shows the top view, in which the same sectors (S1–S8) appear as angular wedges, and the radial dimension is discretised into concentric tracks and finer radial blocks. These two views, combined, create the unique spatial address (L,T,S,B) of each sensor node and offer a basis for local cluster organisation and hierarchical forwarding.

#### 4.2.1. Sector and Block Formation

In each sensor node, an identifier of the spatial block (L,T,S,B) is received. The node identifier is a different unique identifier and is never used as a block identifier. For example, (2,1,3,4) denotes level 2, track 1, sector 3, block 4. This separation allows the distinction of nodes with the same spatial block based on network identifier for medium access control and routing operations.

#### 4.2.2. Radial Tracks

The radial track means that the communication regions are concentric with respect to the cylinder axis. The number of tracks is calculated based on the ratio of deployment radius and the communication range set. For the topology being evaluated, the deployment radius is R=500 m and the communication range is $R_{c}=500$ m, so the entire radial range is covered by a single track:

(11) $$T_{n}=\left\lceil \frac{R}{R_{c}}\right\rceil =1.$$

Thus, the track index does not introduce an additional concentric subdivision in the present experiment. The topology contains one radial track, while the finer radial subdivision is provided by the four blocks defined within each angular sector. If a future experiment uses a smaller communication range, the same definition can generate multiple concentric tracks without changing the remaining (L,T,S,B) indexing principle.

#### 4.2.3. Angular Sectors

The single radial track has been subdivided azimuthally into S=8 equal sectors, each of which spans $45^{\circ }$. The sector index is thus an indication of the angular position of a node around the cylinder axis. This division ensures that all nodes at the same depth and radial distance are not considered to be one large undifferentiated area. Rather, nodes have a bounded angular neighbourhood, so that they can be used for spatial organisation of local clusters and for the storage of routing information in a manageable area. The eight-sector arrangement provides all-around coverage around the cylinder, because $8\times 45^{\circ }=360^{\circ }$. Spatial assignment is based on sector boundaries, which are not assumed to be physical communication barriers.

#### 4.2.4. Vertical Levels

The cylinder can be partitioned into length L=4 sections, each with a height of h=H/L=250 m. Level 1 is the lowest level, and level 4 is the highest level. The level index gives the vertical part of the identifier (L,T,S,B) and is also used to create the hierarchy of cluster heads within the region. A single regional cluster head is located along the axis of the cylinder at the centre depth for each level. Thus, the four regional cluster heads act as intermediate aggregation and forwarding points between the local cluster heads and the surface sink. The level structure is thus not just a geometric description but is also directly related to the routing structure that is evaluated in this study, which is hierarchical. At the lower levels, traffic can be concentrated locally and then sent to the next higher regional cluster head.

#### 4.2.5. Radial Blocks

For each sector, the radial interval is [0,R] and the number of blocks is B=4, with each block having width R/B=125 m, so *B* represents the index of radial block in a sector and not the number of tracks. The finest spatial part of the (L,T,S,B) identifier in the topology evaluated is the block index. Four levels, one track, eight sectors, and four radial blocks give a total of 128 spatial blocks (4×1×8×4=128). Each deployed sensor node is assigned to exactly one of these blocks, while its unique node identifier remains separate from the spatial identifier. This separation is vital, as multiple nodes can be located in the same spatial block while each node has to maintain its own network identifier. The block structure also gives the basis for cluster formation in the local area and for the association of nodes to their cluster head for the information-request and acknowledgement process.

### 4.3. Node Deployment

The deployment algorithm assigns every sensor node to exactly one (L,T,S,B) block. Four regional cluster heads are fixed at the cylinder axis at the centre depth of levels 1–4, and the single surface sink is located at (0,0,H). The regional cluster heads are not treated as gateway nodes. The deployment procedure and the assignment of sensor nodes to their corresponding spatial blocks are summarised in Algorithm 1.
**Algorithm 1** Node deployment and (L,T,S,B) assignment**Input:** Cylinder CR(R,H), total sensor nodes *N*, levels *L*, tracks $T_{n}$, sectors *S*, radial blocks *B*.**Output:** Sensor coordinates, four regional cluster head positions, and surface-sink position. 1:**procedure** NodeDeployment($CR,N,L,T_{n},S,B$) 2:    $h_{l}\leftarrow H/L$, Δθ←2π/S, Δr←R/B 3:    **for** l=1 to *L* **do** 4:        **for** t=1 to $T_{n}$ **do** 5:           **for** s=1 to *S* **do** 6:               **for** b=1 to *B* **do** 7:                   Sample the required number of nodes uniformly inside the corresponding radial, angular, and vertical bounds. 8:                   Assign block identifier (l,t,s,b) to each sampled node. 9:               **end for**10:           **end for**11:        **end for**12:    **end for**13:    **for** l=1 to *L* **do**14:        Place one fixed regional cluster head at $\left(0,0,\left(l-0.5\right)h_{l}\right)$.15:    **end for**16:    Place the single surface sink at (0,0,H).17:**end procedure**

### 4.4. Neighbour Nodes

The set of sensor nodes located within the communication range of node *i* is referred to as its neighbour node set, denoted by $NN_{i}$. Let N denote the set of all sensor nodes in the network, and let $R_{c}$ represent the communication range of each node. The neighbour node set is defined as

(12) $$NN_{i}=\left\{j\in N\;|\;d\left(i,j\right)\leq R_{c},\;j\neq i\right\},$$

where d(i,j) denotes the Euclidean distance between nodes *i* and *j*, computed as

(13) $$d\left(i,j\right)=\sqrt{{\left(x_{i}-x_{j}\right)}^{2}+{\left(y_{i}-y_{j}\right)}^{2}+{\left(z_{i}-z_{j}\right)}^{2}}.$$

The neighbour node set is used during the cluster head selection process to identify the neighbouring nodes of each candidate node and compute its node degree.

### 4.5. Node Degree

Node degree is defined as the number of neighbouring nodes that lie in the communication range $R_{c}$ of a given node. It is expressed as

(14) $$ND_{i}=\sum_{\begin{array}{c} j=1\\ j\neq i \end{array}}^{N}I\;\left(d\left(i,j\right)\leq R_{c}\right).$$

### 4.6. Cluster Centroid Computation

The centroid is computed for the nodes belonging to the same local spatial block or cluster. Since angular coordinates are periodic, the centroid is first calculated in Cartesian coordinates and only then converted to cylindrical form for reporting. For a cluster C containing *k* nodes, the Cartesian centroid is

(15) $${\bar{x}}_{C}=\frac{1}{k}\sum_{j\in C}x_{j},\;{\bar{y}}_{C}=\frac{1}{k}\sum_{j\in C}y_{j},\;{\bar{z}}_{C}=\frac{1}{k}\sum_{j\in C}z_{j}.$$

The corresponding cylindrical representation is

(16) $$CC_{C}=\left(\sqrt{{\bar{x}}_{C}^{2}+{\bar{y}}_{C}^{2}},atan2\left({\bar{y}}_{C},{\bar{x}}_{C}\right),{\bar{z}}_{C}\right).$$

For a candidate node *i*, the centroid-distance criterion is $d_{c,i}={∥\left(x_{i},y_{i},z_{i}\right)-\left({\bar{x}}_{C},{\bar{y}}_{C},{\bar{z}}_{C}\right)∥}_{2}$. To make this criterion comparable with the other benefit criteria, centroid proximity is defined as follows:

(17) $$CC_{i}=1-\frac{d_{c,i}-d_{c,\min}}{d_{c,\max}-d_{c,\min}},$$

where the minimum and maximum are evaluated over the candidate nodes in the same cluster. If all candidates have the same centroid distance, $CC_{i}=1$ for all candidates. Thus, a larger $CC_{i}$ always denotes greater proximity to the cluster centroid. For candidate node *i*, the distance to the block centre is

(18) $$D_{i}={∥\left(x_{i},y_{i},z_{i}\right)-\left({\bar{x}}_{B},{\bar{y}}_{B},{\bar{z}}_{B}\right)∥}_{2},$$

where $\left({\bar{x}}_{B},{\bar{y}}_{B},{\bar{z}}_{B}\right)$ is the geometric centre of the local (L,T,S,B) block.

### 4.7. Network Initialisation Phase

To start the initialisation process, the sink node sends an information request message with the fields shown in Table 2. Upon receiving the information request message, each node generates its block identifier and re-broadcasts the information request message by updating the block identifier field with its own block identifier. This ensures that all nodes have unique block identifiers.

Cluster heads periodically disseminate beacon packets containing several fields, including the cluster head identifier, block identifier, message type, packet sequence number, timestamp, the cluster head’s own coordinates, residual energy, and destination address. To facilitate efficient communication and message dissemination, each cluster head maintains an information table $R_{tab}\left(CH_{i}\right)$. Table 3 presents the packet format for cluster heads.

#### 4.7.1. Acknowledgment Message Procedure

When an information request message is received, each sensor node generates an acknowledgment message that contains its current state information and updates the information in its table to keep track of the communication links that are in progress and the network status. Table 4 presents the acknowledgment message format.

The packet sequence number ensures the proper order of message transmission and update; the sensor node identifier identifies the node that responded; the cluster head identifier specifies the cluster head governing the node; the block identifier indicates the sector or block where the relaying node is located; residual energy reflects the amount of energy the node has remaining; node coordinates indicate the position of the node within the network in three dimensions; and timestamp records the time taken by the node to transmit the message and process the information. This exchange provides a path for routing that allows for logical sectoring across the network. Using the information that has been shared, selected nodes are considered optimal head aggregation nodes. The nodes are then nominated in the network as cluster heads.

#### 4.7.2. Organising Sensor Nodes into Clusters

Only the sink node and the active cluster heads send information request messages. Only the nodes in a specific local cylindrical partition send the information request messages. Table 5 presents the fields of the information request messages sent by each node.

When an information request message is received, a sensor node checks whether the cluster head identifier and block identifier in the information request message match the assigned block identifier and the cluster head identifier of the sensor node. For a match, the sensor node estimates the distance from the cluster head using the received signal strength or Time of Arrival. If this estimated distance lies within the predefined distance threshold, then the node joins the cluster. All other information request messages (received from foreign blocks) are discarded by the source nodes. We describe the detailed process for handling the information request messages in Algorithm 2. Local cluster schedules use Time Division Multiple Access (TDMA) to reduce overlapping transmissions and idle listening [52,53].

**Algorithm 2** Integrated information request message procedure
**Input:** Sink node (SK), cluster head (CH), information request message, $R_{tab}\left(CH_{i}\right)$.**Result:** Communication is initialised, unique node identifiers are assigned, and routing tables are updated.
 1:**procedure** Integrated Information Request Message Procedure(SK,CH, information request message $,R_{tab}\left(CH_{i}\right)$) 2:    **Phase 1: Information request message transmission by sink node** 3:    Construct information request message: 4:            MessageType ← information request message 5:            PacketSequenceNumber ← GetNextSequenceNumber() 6:            Sink node identifier ← GetSourceNodeIdentifier() 7:            DestinationAddress ← BROADCAST 8:            NodeCoordinates ← GetSourceNodeCoordinates() 9:            Timestamp ← GetCurrentTime()10:    Broadcast an information request message.11:    Each sensor node receives the information request message and assigns a unique node identifier.12:    **if** rebroadcast is required (no acknowledgment received) **then**13:        Increment BlockIdentifier.14:        Update information request message.BlockIdentifier.15:        Rebroadcast the updated information request message.16:        Update node information.17:    **end if**18:    **Phase 2: Cluster head beacon transmission**19:    **if** flag(x)=0 **then**20:        Construct beacon packet (BP):21:                MessageType ← Beacon22:                PacketSequenceNumber ← GetNextSequenceNumber()23:                Cluster head identifier ← GetClusterHeadIdentifier()24:                BlockIdentifier ← GetSourceBlockIdentifier()25:                DestinationAddress ← BROADCAST26:                NodeCoordinates ← GetClusterHeadCoordinates()27:                Timestamp ← GetCurrentTime()28:                ResidualEnergy ← GetClusterHeadResidualEnergy()29:        Broadcast BP.30:        **if** Beacon is received from a neighbouring cluster head **then**31:           Update $R_{tab}\left(CH_{i}\right).$Table.32:           Store neighbouring cluster head information.33:           Set flag(x)←1.34:           Reset flag(x)←0 after processing.35:        **end if**36:    **end if**37:    **Phase 3: Acknowledgment message processing**38:    Construct acknowledgment:39:            MessageType ← Acknowledgment40:            PacketSequenceNumber ← GetPacketSequenceNumber()41:            Sensor node identifier ← GetSourceNodeIdentifier()42:            BlockIdentifier ← GetSourceBlockIdentifier()43:            NodeCoordinates ← GetNodeCoordinates()44:            Timestamp ← GetCurrentTime()45:            ResidualEnergy ← GetResidualEnergy()46:    Broadcast acknowledgment.47:    **if** information request message is received by sensor node $S_{i}$ **then**48:        Discard the information request message.49:    **else if** information request message is received by cluster head $CH_{i}$ **then**50:        Update $R_{tab}\left(CH_{i}\right).$Table.51:        **for** each non-cluster-head node $N_{i}$ **do**52:           Update $R_{tab}\left(N_{i}\right).$Table using PacketSequenceNumber and BlockIdentifier.53:        **end for**54:    **end if**55:
**end procedure**



#### 4.7.3. Cluster Head Selection

The proposed protocol uses an MCDM score to rank candidate nodes in each local block. Four criteria are considered: node degree, residual energy, centroid proximity, and distance to the block centre. Node degree and residual energy are benefit criteria, centroid proximity is converted into a benefit score in Equation (17), and distance to the block centre is a cost criterion.

The benefit criteria are normalised by min–max scaling [54] as follows:

(19) $$\begin{array}{cc} ND_{i}^{norm} & =\frac{ND_{i}-ND_{\min}}{ND_{\max}-ND_{\min}}, \end{array}$$

(20) $$\begin{array}{cc} RE_{i}^{norm} & =\frac{RE_{i}-RE_{\min}}{RE_{\max}-RE_{\min}}, \end{array}$$

while the distance-to-block-centre criterion is normalised as follows:

(21) $$D_{i}^{norm}=\frac{D_{i}-D_{\min}}{D_{\max}-D_{\min}}.$$

If the range of any criterion is zero, all candidates receive a value of one for that criterion because no candidate can be distinguished by that criterion in that block. The resulting decision score is

(22) $$F\left(V,CH_{i}\right)=\alpha ND_{i}^{norm}+\beta RE_{i}^{norm}+\varepsilon CC_{i}+\delta \left(1-D_{i}^{norm}\right),$$

where

(23) α+β+ε+δ=1.

The weighted-sum decision rule is adopted to fulfil the general principles of MCDM [37,55]. The configuration evaluated gives node degree, residual energy, centroid proximity, and distance to the block centre weights of α=0.25, β=0.35, ε=0.20, and δ=0.20, respectively. These weights add up to one, and the weight of residual energy is the highest individual weight because it directly affects the ability of a candidate node to support forwarding. The selection score does not rely only on residual energy, as it also includes the contributions from node connectivity and spatial scores. The sensitivity experiment is used to study the effect of changing the value of β and to find the value of β=0.35 that gives the best observed packet delivery ratio and network lifetime. The result of this experiment is valid for the investigated weight configuration and does not prove the global optimality of the complete weight vector, since the other weights were not rebalanced. The candidate with the highest $F(V,CH_{i})$ is elected as the cluster head for the corresponding local block. Cluster head roles are rotated when residual energy falls below 20% of the initial energy. The local ranking requires O(nm) criterion evaluations for *n* candidates and m=4 criteria. All of the above steps are summarised in Algorithm 3.

**Algorithm 3** Network division, cluster head selection, and cluster formation
**Input:** Network zone $NZ(R,H,R_{c})$, sensor nodes *N*, sector count *S*, distance threshold DT.**Output:** Cluster heads, cluster members, TDMA schedule.
 1:**procedure** ClusterFormation(NZ,N)

**Phase 1: Network division**

 2:    Initialise block list Blocks←∅ 3:    Compute number of tracks $T_{n}\leftarrow \left\lceil R/R_{c}\right\rceil$ and levels L←H/h 4:    **for** each level **do** 5:        **for** each track **do** 6:           Compute the radial block boundaries 7:           **for** each sector and radial block **do** 8:               Assign block identifier (L,T,S,B) 9:           **end for**10:        **end for**11:    **end for**

**Phase 2: Cluster head selection**

12:    **for** each sensor node i∈N **do**13:        Compute residual energy $RE_{i}$ using Equation (10)14:        Compute node degree $ND_{i}$ using Equation (14)15:        Compute the Cartesian cluster centroid using Equation (15) and the centroid-proximity score $CC_{i}$ using Equation (17)16:        Compute distance $D_{i}$ from the block centre using Equation (18)17:        Normalise the four criteria and evaluate $F(V,CH_{i})$ using Equation (22)18:    **end for**19:    Elect the node with the highest $F_{i}$ in each block as the cluster head20:    Assign remaining nodes as cluster members

**Phase 3: Cluster formation**

21:    **for** each cluster head $CH_{i}$ **do**22:        Broadcast beacon message B.MSG23:    **end for**24:    **for** each sensor node **do**25:        Receive beacon message26:        **if** Block identifiers match and $d(i,CH_{i})\leq DT$ **then**27:           Send join-request to $CH_{i}$28:           Update cluster membership29:        **end if**30:    **end for**

**Phase 4: Cluster registration**

31:    **for** each cluster head **do**32:        Broadcast information-request message33:    **end for**34:    **for** each cluster member **do**35:        Receive information-request message36:        Select the eligible nearest cluster head37:        Send acknowledgement message38:    **end for**39:    **for** each cluster head **do**40:        Register cluster members from acknowledgement messages41:    **end for**

**Phase 5: TDMA scheduling**

42:    **for** each cluster head **do**43:        Allocate TDMA slots44:        Broadcast TDMA schedule45:    **end for**46:    **for** each cluster member **do**47:        Transmit sensed data during the allocated TDMA slot48:    **end for**49:
**end procedure**



### 4.8. Reliable Data Transmission

Our proposed protocol enables reliable data transmission in three different modes, as Algorithm 4 shows.

**Algorithm 4** Reliable data transmission
**Input:** Sensor node (SN), local cluster head (CH), regional cluster head (RCH), routing tables, current layer, and forwarding threshold.**Output:** Application data delivered to the single surface sink or discarded when no eligible next hop exists.
 1:**procedure** ReliableDataTransmission(SN,CH,RCH) 2:    **Phase 1: Sensor node to local cluster head** 3:    Retrieve the current node position, block identifier, and assigned cluster head identifier. 4:    **if** CH is within the cluster-joining distance and belongs to the assigned local block **then** 5:        Transmit the application packet from SN to CH. 6:    **else** 7:        Select the nearest eligible cluster head within the communication range. 8:        **if** an eligible cluster head exists **then** 9:           Forward the packet to the selected cluster head.10:        **else**11:           Discard the packet and record a forwarding failure.12:        **end if**13:    **end if**14:    The local cluster head aggregates/fuses the received application data.15:    **Phase 2: Local cluster head to regional cluster head**16:    **for** each active local cluster head $CH_{i}$ **do**17:        Determine the designated regional cluster head for the node’s level.18:        **if** $CH_{i}$ can reach its designated RCH within the configured range **then**19:           Transmit the aggregated packet to the designated RCH.20:        **else**21:           Select an eligible regional cluster head that provides upward progress.22:           **if** an eligible RCH exists **then**23:               Forward the aggregated packet to the selected RCH.24:           **else**25:               Discard the packet and record a forwarding failure.26:           **end if**27:        **end if**28:    **end for**29:    **Phase 3: Regional cluster head hierarchy to surface sink**30:    **for** each $RCH_{i}$ **do**31:        Determine the current level of $RCH_{i}$.32:        **if** $RCH_{i}$ is at level 4 **then**33:           Transmit the aggregated packet directly to the single surface sink.34:        **else**35:           Select the next upper-level regional cluster head.36:           **if** the next RCH satisfies the forwarding eligibility condition **then**37:               Forward the packet to the selected upper-level RCH.38:           **else**39:               Discard the packet and record a forwarding failure.40:           **end if**41:        **end if**42:    **end for**43:
**end procedure**



#### 4.8.1. Data Transmission from Sensor Node to Cluster Heads

Member nodes send data to their cluster heads. Every sensor node searches its routing table to identify the allocated cluster head for the data. If the sensor node’s block identifier matches the block identifier of its allocated cluster head, the sensor node forwards the data to the allocated cluster head; otherwise, it forwards the data to the nearest available cluster head. Upon receiving the data, each cluster head performs merging and fusion operations [56] to eliminate redundant packets and reduce transmission overhead.

#### 4.8.2. Data Transmission from Cluster Head to Regional Cluster Head

Regional cluster heads collect the aggregated data from regular cluster heads. A cluster head that is within one-hop range of a regional cluster head forwards the data directly to that regional cluster head. A cluster head that is not within one-hop range of any regional cluster head looks up its routing table to find the nearest regional cluster head. The cluster head must confirm that its block identifier matches the block identifier of the target regional cluster head before forwarding the data to avoid a forwarding loop.

#### 4.8.3. Data Transmission from the Regional Cluster Head to the Surface Sink

Regional cluster heads perform multi-hop communication toward the single surface sink to forward the aggregated data. At level 4, the regional cluster head forwards the data to the surface sink directly. Regional cluster heads at levels 1–3 forward data through the next higher-level regional cluster head to reliably and efficiently forward the data across the multi-layer hierarchical network structure.

An intermediate forwarding regional cluster head is selected based on the layer identifier in ascending order. Table 6 includes the regional cluster head identifier and the layer number as routing information in a data packet. Given the bottom–up multi-level routing approach, the number of hops from a regional cluster head to the surface sink depends on its level. Under the stated hierarchy, a packet forwarded from the level 1 regional cluster head traverses the level 2, level 3, and level 4 regional cluster heads before reaching the sink, giving four links from the level 1 regional cluster head to the sink. Packets originating at higher levels traverse fewer links. An average hop count depends on the traffic distribution and the measured routing traces and is not inferred from the number of levels alone. Hop count affects delay and energy, as we discuss in Section 6.

### 4.9. Interference Management Between Clusters

In the IoUT, simultaneous transmissions by neighbouring clusters can cause packet collisions and degrade QoS. QEE-CBR coordinates intra-cluster transmissions using TDMA scheduling at each cluster head, organises nodes spatially through cylindrical partitioning, and structures inter-cluster forwarding through regional cluster heads. These mechanisms reduce contention within and across clusters, and adding a network-wide inter-cluster scheduling mechanism or an explicitly modelled interference process represents a natural next step toward even stronger collision-avoidance guarantees.

## 5. Simulation Work

This section describes the simulation environment and configuration used for the evaluation. Section 6 presents the numerical results, sensitivity experiments, ablation study, and the application context of the protocol.

### 5.1. Selection of Baseline Schemes

We compared the proposed QEE-CBR protocol against four main IoUT routing protocols, namely, BEEC, MLCEE, RQAR, and EAVARP. These schemes represent balanced energy-efficient clustering, multi-level clustering, QoS-aware forwarding without hierarchical clustering, and void-aware forwarding, respectively. We also included FNCBR [40] as a targeted architectural comparator, while QE-ORP [20] was evaluated separately because it uses a flat cooperative opportunistic architecture rather than hierarchical clustering.

This work evaluates the four main benchmark protocols under a common set of stated network and traffic conditions: node-density configurations, deployment region, packet size, application generation rate, propagation model, communication range, initial sensor node energy, retransmission limit, queue size, and simulation horizon. The protocol-specific routing and cluster-selection mechanisms are retained for each benchmark. No QEE-CBR-specific cylindrical partitioning, MCDM score, regional cluster head hierarchy, or aggregation rule is inserted into a baseline.

### 5.2. Simulation Environment and Configuration

We evaluated the proposed protocol and the four main benchmark protocols using the NS-3 discrete-event network simulator, version ns-3.40 [57]. We executed the simulations on a MacBook Pro equipped with an Apple M1 processor, 16 GB of random-access memory, and a 512 GB solid-state drive, running macOS Monterey; NS-3 was compiled using the GNU Compiler Collection (g++) version 11.2.0. The host-computer specifications were for execution only and were not used as acoustic-node power parameters. The underwater acoustic configuration was inspired by representative LinkQuest UWM1000 modem characteristics [58]. The amplifier energy coefficient, ambient noise model, and SNR detection threshold used by the channel model are also listed in Table 7. A fixed pseudo-random seed was used for the topology, mobility, and traffic processes.

### 5.3. Measurement Procedures

We executed each simulation run for 2500 s, which is longer than the largest observed first-node-death time. Network lifetime is defined as the time from network start until the first sensor node exhausts its available energy. Packet delivery ratio, received throughput, end-to-end delay, energy consumption, hop count, and the targeted robustness/sensitivity results were collected according to the definitions in Section 5.5. The same topology dimensions, traffic generation, acoustic propagation model, energy model, simulation duration, and measurement definitions were applied to the four main benchmark protocols. Protocol-specific clustering, forwarding, void-handling, or mobile-sink mechanisms were retained according to the respective benchmark logic rather than replaced by QEE-CBR mechanisms. FNCBR and QE-ORP were used only in the targeted architectural comparisons described later, because their topology and forwarding assumptions differ from the four main benchmark configurations.

The evaluation follows network initialisation, spatial assignment, neighbour discovery, cluster formation, MCDM-based cluster head selection, local scheduling, data transmission and aggregation, hierarchical forwarding, and metric collection. When the cluster head rotation condition is met, the affected local cluster is re-evaluated using the same selection procedure. The single surface sink is the final acoustic destination for underwater traffic. There are 128 blocks in the partition, so at lower node densities, some blocks may be empty. Cluster head selection is applied only to occupied blocks.

### 5.4. Simulation Setup

The network region has 1000m×1000m×1000m dimensions. The actual occupied underwater region is the right circular cylinder with radius 500 m and height 1000 m. The node-density sweep of 100–500 sensors is deployed in the network region. Sensor nodes use the configured random-walk mobility model [59] with speeds of 2–3 m/s, whereas the four regional cluster heads and the single surface sink remain fixed. The mobility setting serves as a controlled stress condition and can be complemented by additional mobility patterns in future work. Node positions used by routing are obtained from the stated Time-of-Arrival-based localisation procedure [42,60]. Current-driven drift and broader mobility models are discussed in the limitations and future work sections of this paper. Table 7 presents the key execution, topology, traffic, acoustic, and routing parameters.

### 5.5. Performance Metrics

We evaluated the performance of the proposed protocol using six metrics that collectively capture reliability, efficiency, and load-balancing capability.

#### 5.5.1. Average Packet Delivery Ratio

The average packet delivery ratio is defined at the application-packet level, using the unique source packet identifier to avoid counting retransmissions or aggregated forwarding copies as additional source packets:

(24) $$\mathrm{PDR}=\frac{N_{\mathrm{delivered}}}{N_{\mathrm{generated}}}\times 100\%,$$

where $N_{\mathrm{generated}}$ is the number of unique application packets generated by the sensor nodes during the measurement interval, and $N_{\mathrm{delivered}}$ is the number of those original application packets successfully delivered to the single surface sink. Retransmitted copies and intermediate aggregated packets are not counted as additional application packets.

#### 5.5.2. Average Network Throughput

Network throughput is defined as the rate of application-payload bytes represented by data delivered to the single surface sink, rather than a count of retransmitted bytes. It is expressed in kbps:

(25) $$\mathrm{NTP}=\frac{8B_{\mathrm{recv}}}{T_{\mathrm{obs}}\times 1000},$$

where $B_{\mathrm{recv}}$ is the application payload represented by data delivered to the sink during the observation interval, and $T_{\mathrm{obs}}$ is the interval in seconds. The factors of eight and one thousand convert bytes per second to kbps.

Since aggregation can combine multiple source records, this application-data metric is distinct from the number of bytes physically transmitted over the acoustic channel. The application generation process in this study is defined at the level of active source clusters (occupied (L,T,S,B) blocks) rather than per individual sensor node.

#### 5.5.3. Average End-to-End Delay

The average end-to-end delay is calculated from the source and sink timestamps used by the evaluation:

(26) $$D_{\mathrm{avg}}=\frac{1}{N_{\mathrm{delivered}}}\sum_{i=1}^{N_{\mathrm{delivered}}}\left(T_{i}^{\mathrm{recv}}-T_{i}^{\mathrm{send}}\right),$$

where $T_{i}^{\mathrm{recv}}$ and $T_{i}^{\mathrm{send}}$ are the receive and send timestamps associated with delivered item *i*, and $N_{\mathrm{delivered}}$ is the number of delivered items included in the calculation.

#### 5.5.4. Average Network Lifetime

The network lifetime is defined as the time from the start of network operation until the first sensor node runs out of energy:

(27) $$T_{\mathrm{NL}}=T_{\mathrm{sim}}^{\mathrm{first}/\mathrm{dead}},$$

where $T_{\mathrm{sim}}^{\mathrm{first}/\mathrm{dead}}$ is the simulation time at which the first node’s energy reaches zero. This measure indicates how well the protocol uses an appropriate amount of energy while maintaining a longer network lifetime.

#### 5.5.5. Total Energy Consumption

Total energy consumption is defined as the cumulative energy dissipated by all sensor nodes in the network over the entire simulation period. This includes three distinct components: energy consumed for transmitting data packets, $E_{tx}$; energy consumed for receiving data packets, $E_{rx}$; and energy consumed for data aggregation at cluster heads, $E_{DA}$. The individual components are further described in Section 3 (Equations (7)–(9)). The total energy consumption is computed as follows:

(28) $$E_{\mathrm{total}}=\sum_{n=1}^{N}\left(E_{tx}\left(n\right)+E_{rx}\left(n\right)+E_{DA}\left(n\right)\right),$$

where *N* is the total number of sensor nodes in the network. For non-cluster-head sensor nodes, $E_{DA}\left(n\right)=0$, because data aggregation is performed only at cluster heads; for cluster heads, $E_{DA}\left(n\right)$ is computed using Equation (9) over the aggregated packets. The four fixed regional cluster heads are assumed to be externally powered, and their energy consumption is not included in the sensor-node aggregate metric.

## 6. Results, Analysis, and Applications

This section presents the primary performance results of QEE-CBR, followed by the diagnostic, sensitivity, and ablation analyses. Finally, we also discuss the targeted applications of the proposed protocol. The main performance metrics compare QEE-CBR with BEEC, MLCEE, RQAR, and EAVARP; QE-ORP appears only in the explicitly labelled comparison with our previous work.

### 6.1. Average Packet Delivery Ratio

**Figure 4:**
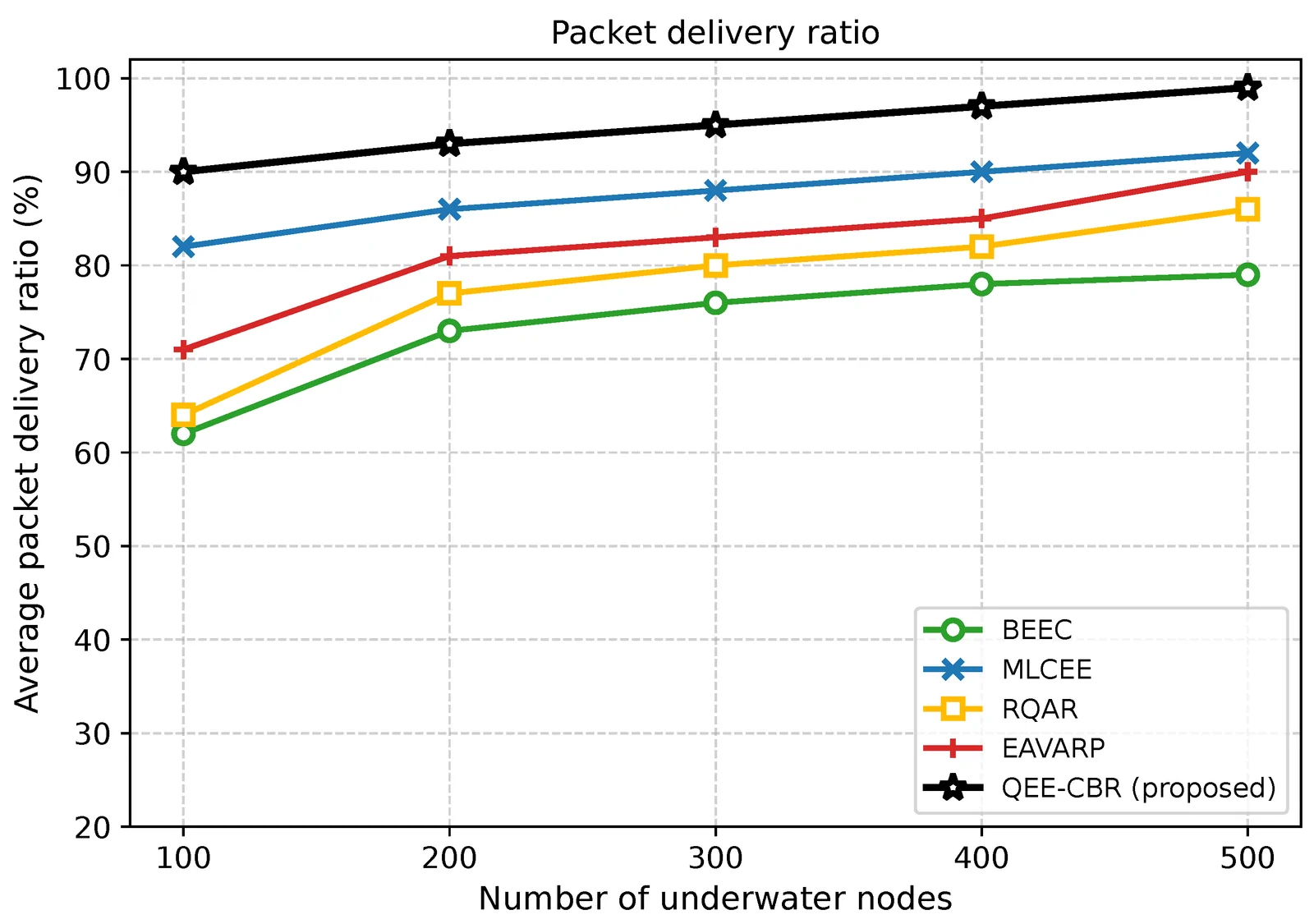
Average packet delivery ratio versus number of underwater nodes.

The packet delivery ratio increases with node density for all five main protocols, as Figure 4 shows. QEE-CBR rises from 90% at 100 nodes to 99% at 500 nodes, whereas MLCEE reaches 92% at 500 nodes.

At 500 nodes, QEE-CBR achieves a 99% packet delivery ratio, compared with BEEC (79%), MLCEE (92%), RQAR (86%), and EAVARP (90%). The proposed protocol therefore maintains the highest delivery ratio in the evaluated density range, with the difference becoming particularly clear at the largest network size. The outcome is consistent with the protocol architecture: local sensor traffic is first confined to a cluster; cluster heads aggregate the information; and the aggregated information is sent out through the regional hierarchy without having to be sent directly by every sensor node that has information to send. Residual energy is not the only factor taken into account in the MCDM selection; node degree, centroid proximity, and distance to the block centre are also considered. QEE-CBR can maintain connectivity and spatial compactness with an increasing number of nodes.

The increase in packet delivery ratio as the number of nodes increases is due to the improvement in the local connectivity and availability of neighbour forwarding candidates in the configuration under evaluation. Density also results in increased traffic and contention, whereas QEE-CBR has 99% at 500 nodes, demonstrating that the hierarchical organisation and aggregation can compensate for the increased offered traffic for the tested NS-3 conditions.

### 6.2. Average Network Throughput

**Figure 5:**
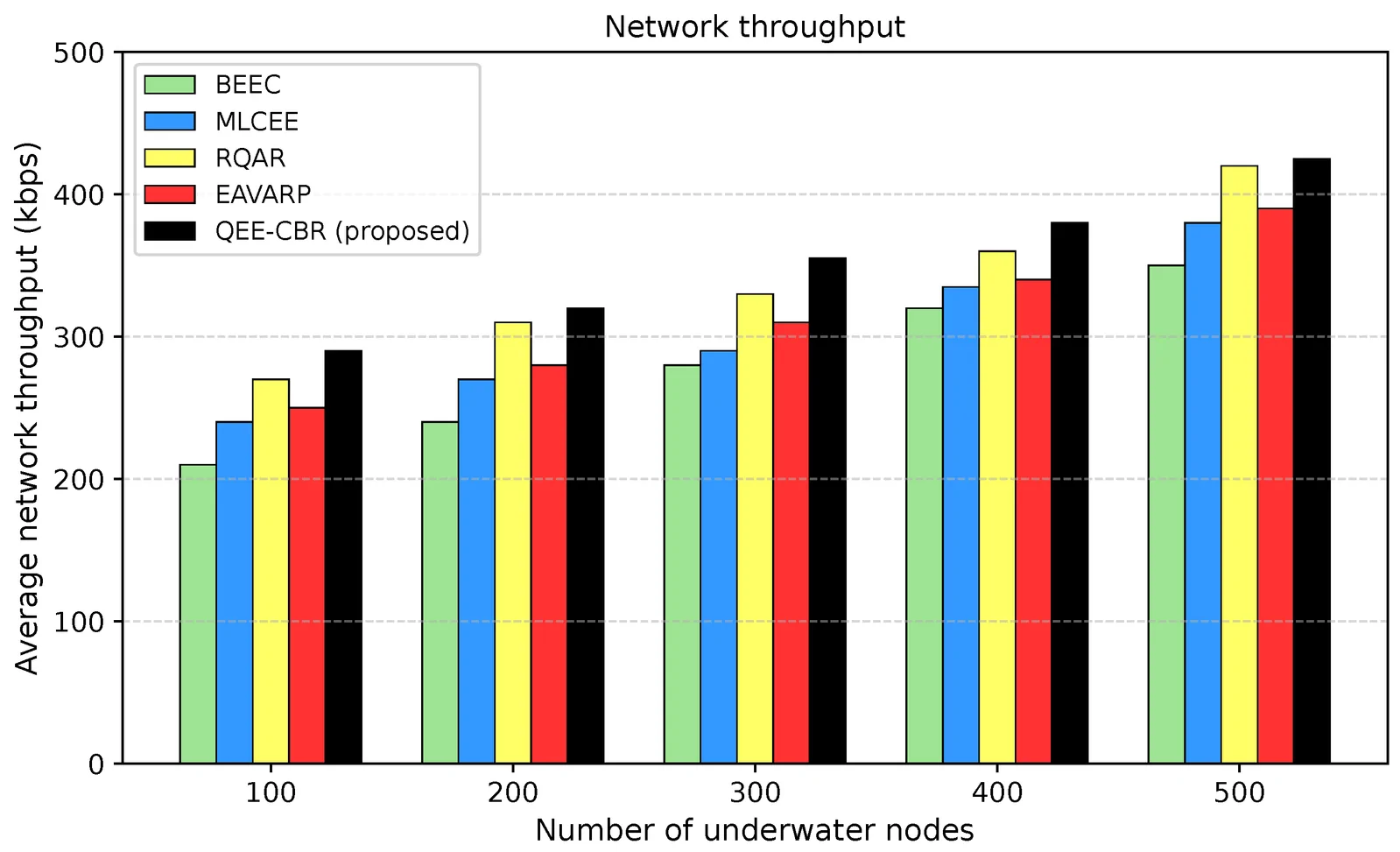
Average network throughput versus number of underwater nodes.

Figure 5 shows the sink-side application-data rate. QEE-CBR increases from 290 kbps at 100 nodes to 425 kbps at 500 nodes.

The metric is defined in Section 5.5 as the rate of application data represented at the sink. Because aggregation can combine multiple source records, this value is distinct from the number of bytes physically transmitted over the acoustic channel. The generation process is defined at the level of active source clusters (occupied (L,T,S,B) blocks), and the value therefore represents the aggregate application-data rate delivered to the sink under the evaluated configuration. At 500 nodes, QEE-CBR gives 425 kbps, compared with BEEC (350 kbps), MLCEE (380 kbps), RQAR (420 kbps), and EAVARP (390 kbps).

### 6.3. Average End-to-End Delay

**Figure 6:**
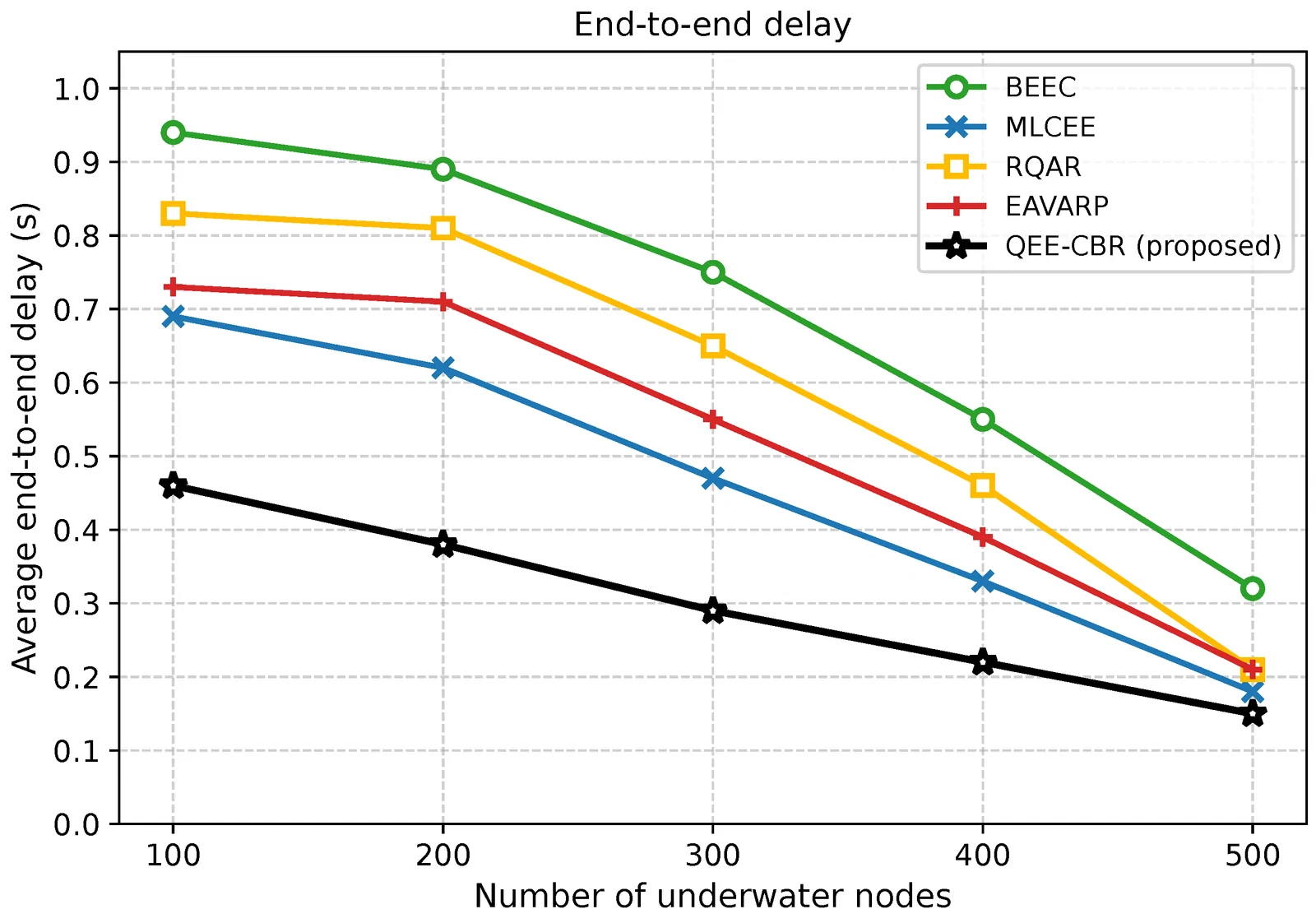
Average delay versus number of underwater nodes.

Figure 6 presents the delay trend. The delay decreases as node density increases. The hop-count results do not show a corresponding reduction in hops; however, this trend is interpreted descriptively and is not attributed conclusively to reduced waiting, retransmission, or physical propagation time.

At 500 nodes, the plotted values are BEEC (0.32 s), MLCEE (0.18 s), RQAR (0.21 s), EAVARP (0.21 s), and QEE-CBR (0.15 s). Analysis of the corresponding hop count and connectivity is reported in the dedicated analysis subsection. The values determine the order of the delay measured for the configuration being evaluated.

### 6.4. Average Network Lifetime

**Figure 7:**
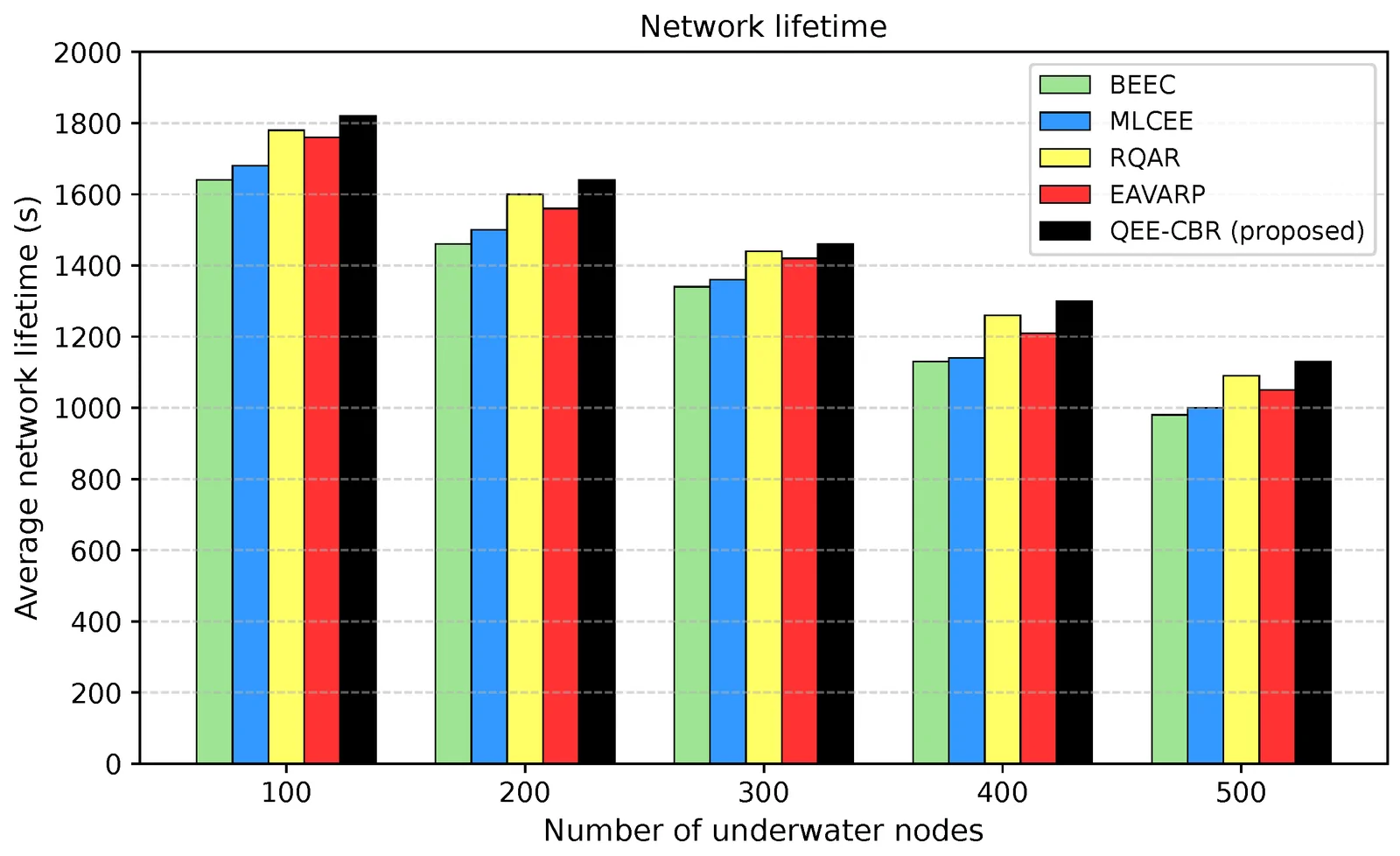
Average network lifetime versus number of underwater nodes.

Figure 7 presents the network lifetime obtained from the 2500 s simulation runs. The lifetime is obtained directly from the duration of the network until the first node is depleted of energy in the common simulation time.

At 100 nodes, QEE-CBR reaches 1820 s, compared with BEEC (1640 s), MLCEE (1680 s), RQAR (1780 s), and EAVARP (1760 s). At 300 nodes, QEE-CBR reaches 1460 s. At 500 nodes, QEE-CBR reaches 1130 s, whereas BEEC reaches 980 s, MLCEE 1000 s, RQAR 1090 s, and EAVARP 1050 s. As the node density increases, the network lifetime decreases as expected, because when the number of nodes increases, the aggregate communication workload also increases, which results in increased energy consumption. The first node will thus exhaust its energy before the second node. QEE-CBR has the best lifetime at all of the densities considered.

The observed gained lifetime is comparable with the use of local aggregation, the shorter communication distances between the cluster head and the members, and the rotation of the ordinary cluster head roles when the members’ residual energy is below the defined threshold. The mechanisms yield a more even distribution of communication work than a design where a single set of forwarding nodes is responsible for the work throughout the experiment.

### 6.5. Total Energy Consumption

**Figure 8:**
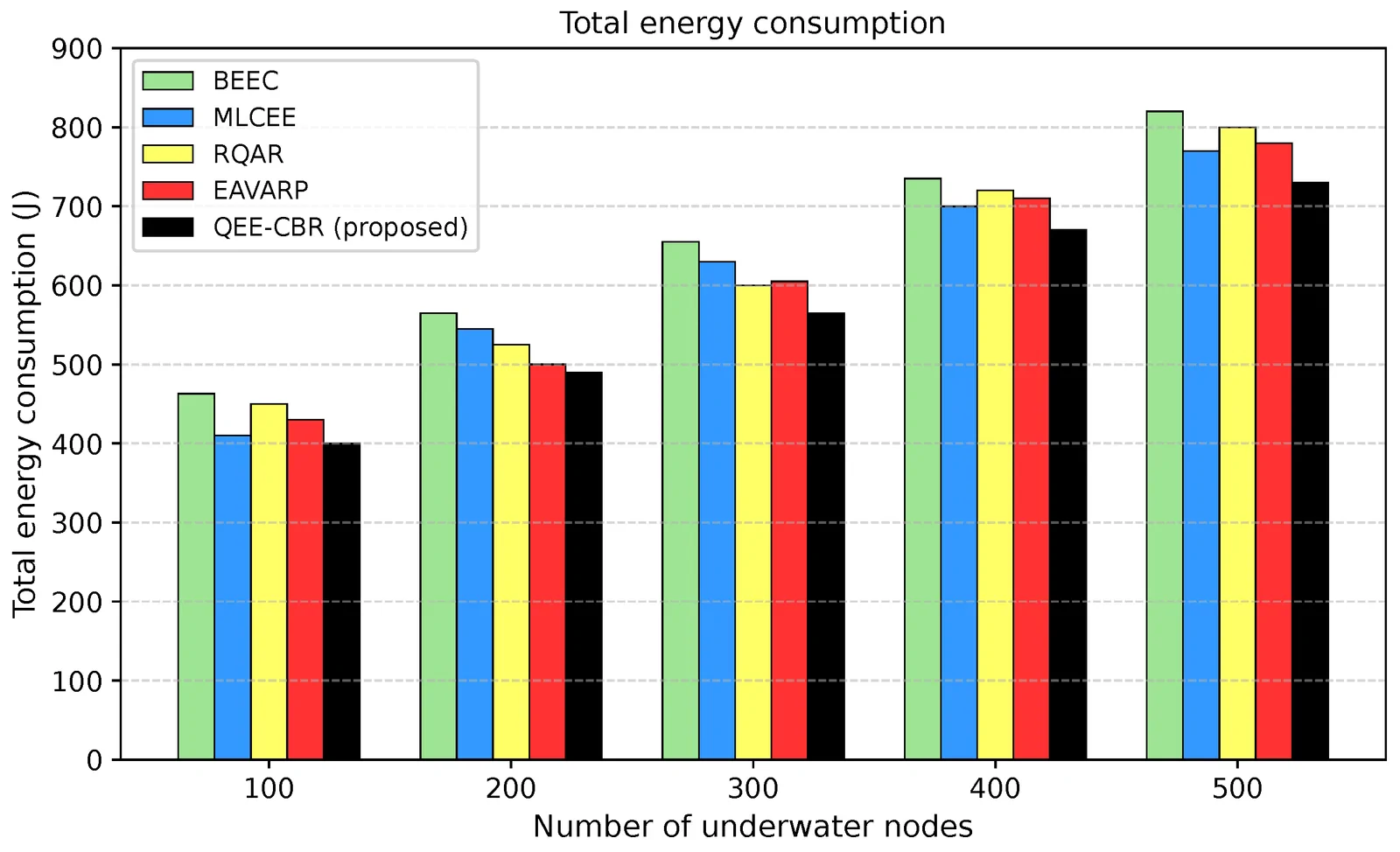
Total energy consumption versus number of underwater nodes.

Figure 8 shows the total energy consumption of the system. QEE-CBR yields the lowest total energy consumption for all of the densities evaluated for the four main benchmarks.

At 500 nodes, QEE-CBR consumes 730 J, compared with BEEC (820 J), MLCEE (770 J), RQAR (800 J), and EAVARP (780 J). This is consistent with aggregation, shorter links from the members to the cluster heads, and hierarchical forwarding. The interpretation of the energy consumption and network lifetime can usefully be made jointly: first, the spatial clustering and aggregation reduce the number of repeated transmissions; second, the MCDM-based cluster head selection and rotation minimise the forwarding overhead among nodes.

The difference is also meaningful because the network becomes increasingly traffic-intensive as the node density rises. A protocol that preserves delivery performance at higher density but consumes substantially more energy would have limited practical benefit for battery-powered underwater nodes. QEE-CBR maintains the lowest total energy among the four main benchmarks in the comparison, while also achieving the highest packet delivery ratio and longest lifetime.

### 6.6. Hop Count and Connectivity Analysis

**Figure 9:**
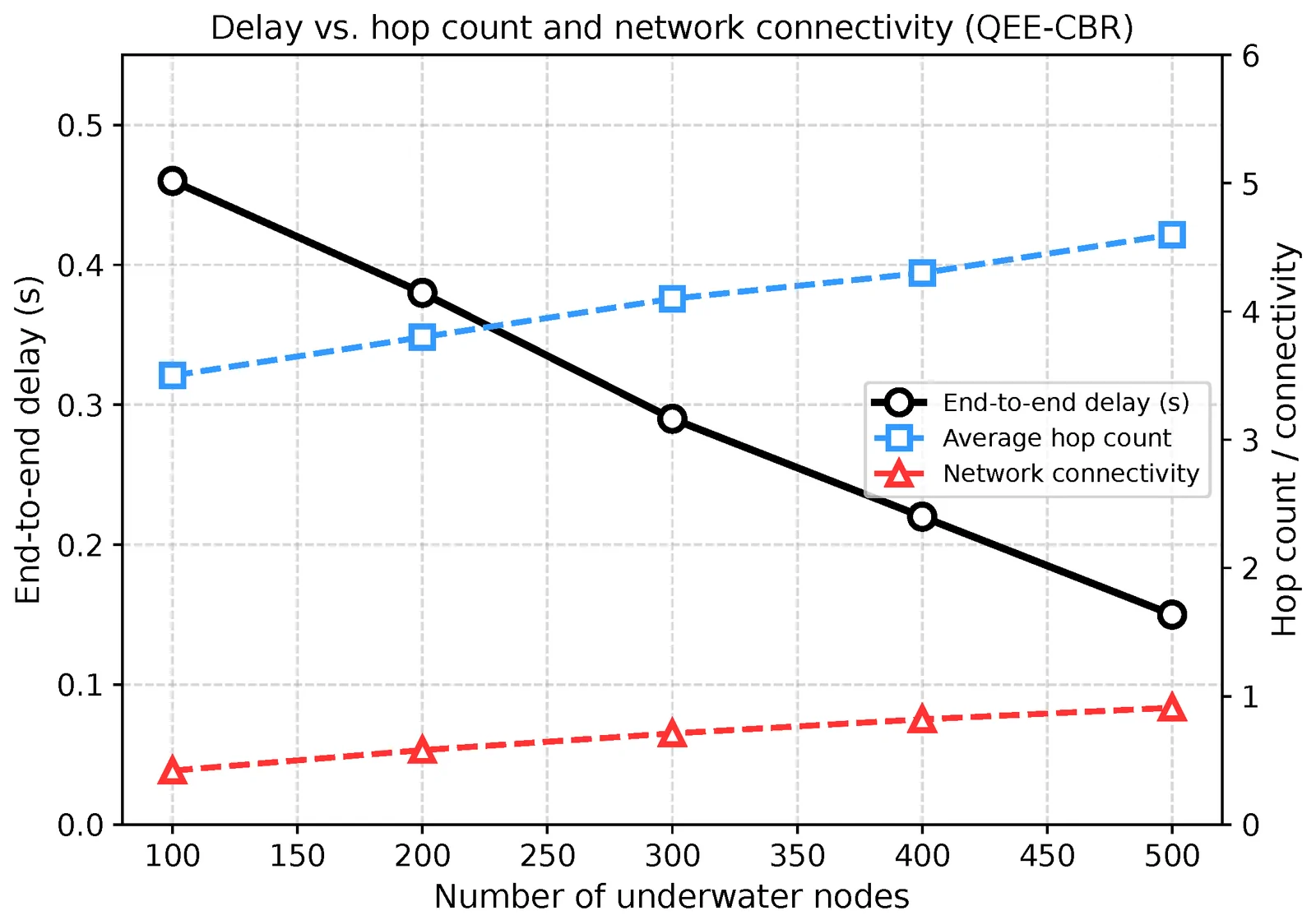
QEE-CBR end-to-end delay, average hop count, and network connectivity versus number of underwater nodes.

Figure 9 presents the dedicated hop-count and connectivity diagnostic after the primary performance results. The figure shows that the mean QEE-CBR hop count increases gradually with node density, while network connectivity also improves. The reduction in end-to-end delay is therefore not attributable to fewer hops. Instead, the higher availability of eligible neighbours and shorter local forwarding distances can reduce waiting and retransmission-related delay sufficiently to offset the additional forwarding stages.

The result can be interpreted in the context of the (L,T,S,B) partitioning and regional cluster head hierarchy. Local clustering limits the spatial scope of member-to-cluster-head communication, while regional cluster heads provide structured upward forwarding. As density increases, a source has more potential neighbours within its local communication region. This improves route availability and reduces unsuccessful forwarding attempts, even though the resulting paths may contain slightly more forwarding stages. The figure therefore provides a topology-level explanation for the decreasing delay trend observed in Figure 6.

For cross-protocol context, Table 8 presents the hop-count comparison. The table is a complementary diagnostic and is not used to recalculate the delay curves.

### 6.7. Performance Under Localisation Noise

We evaluated localisation robustness by adding Gaussian perturbations with σ=10 m to node coordinates. Table 9 gives the resulting performance at 500 nodes, with the noise-free values from Section 6 used as the baseline for the percentage changes shown in parentheses. This is a controlled robustness experiment rather than a claim that Gaussian noise fully represents underwater localisation uncertainty; multipath and refraction can produce more complex errors [61].

Table 9 shows the values for the 10 m localisation-noise experiment. The noise-free and perturbed data have been recalculated as relative changes. For QEE-CBR, the packet delivery ratio decreases from 99.0% to 96.0% (a 3.0% relative decrease, or 3.0 percentage points), delay increases from 0.15 s to 0.16 s (6.7%), energy consumption increases from 730 J to 753 J (3.2%), and throughput decreases from 425 to 413 kbps (2.8%). Across these metrics, there are relatively small changes.

The relative robustness is in line with the fact that the decision for the cluster head is not based on a single geometric quantity. Even with perturbation of centroid and distance estimates, the residual energy and node degree are still included in the MCDM score. Localisation noise therefore affects spatial criteria without forcing the selection process to depend entirely on the distorted position estimate. This experiment offers the opportunity to perform a sensitivity analysis with Gaussian perturbation and can be expanded to a complete underwater localisation model including multipath, refraction, correlated errors, and Doppler effects.

### 6.8. Sensitivity Analysis

**Figure 10:**
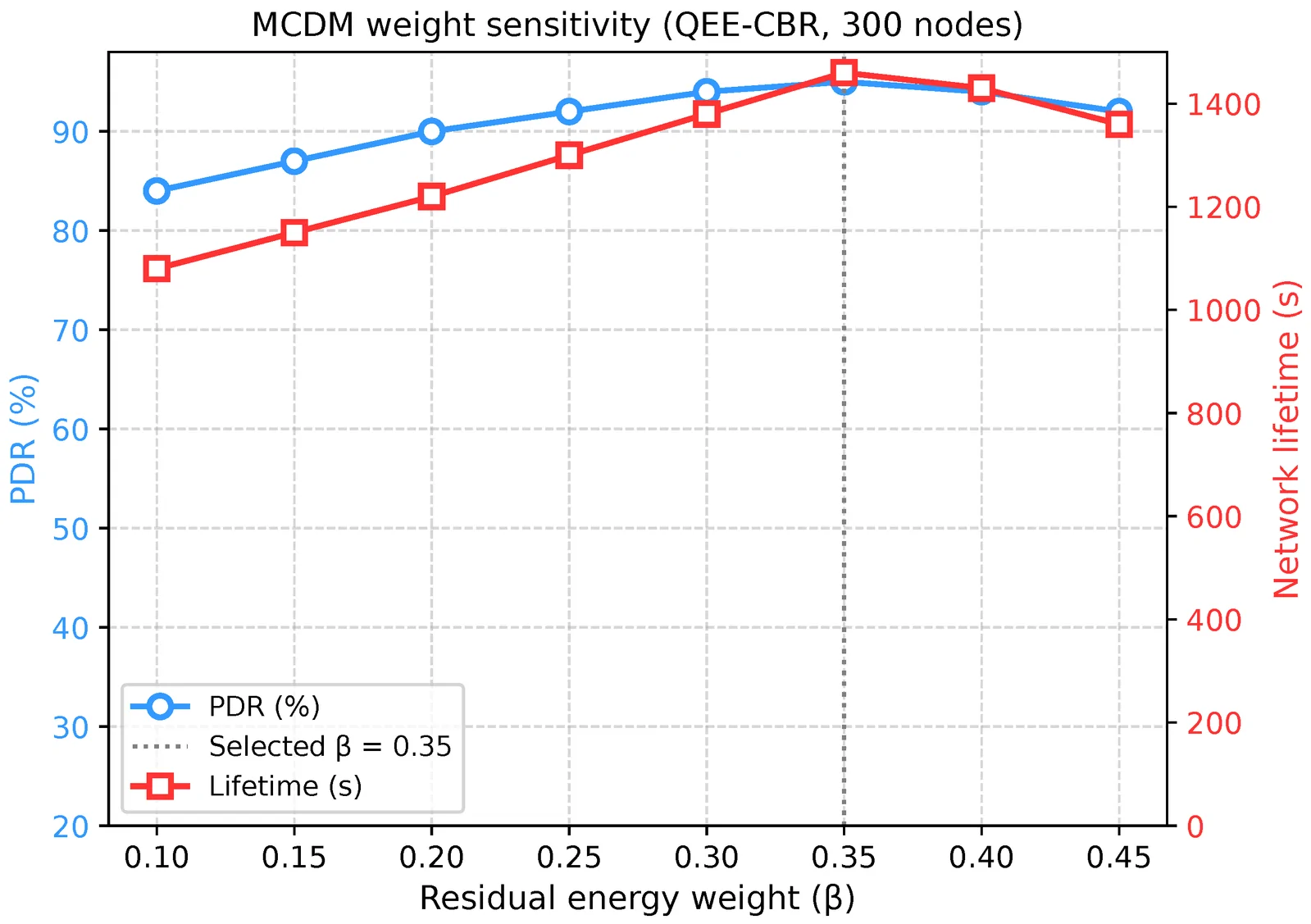
Sensitivity of packet delivery ratio and network lifetime to the residual-energy MCDM weight (β) at 300 nodes.

**Figure 11:**
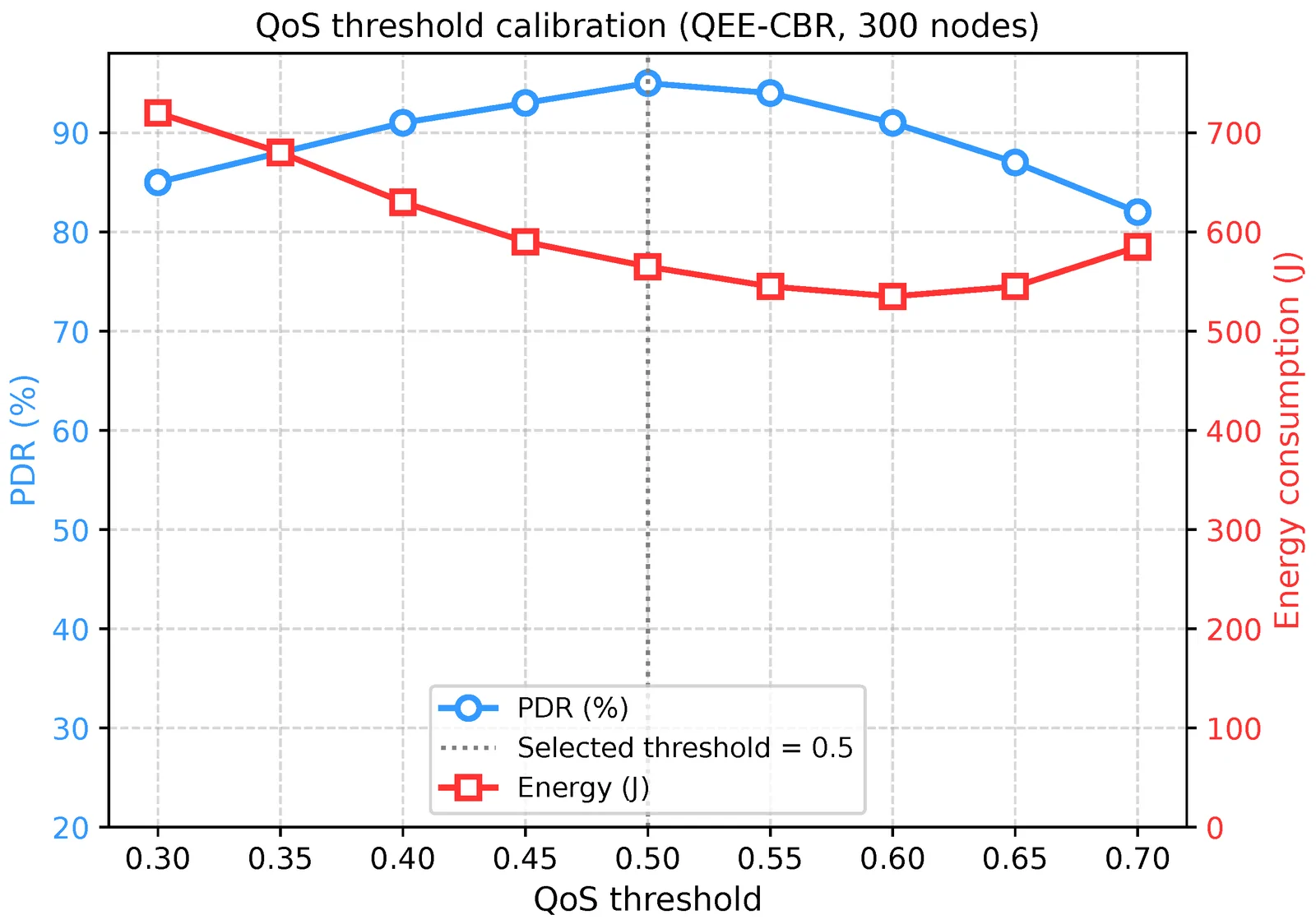
QoS-threshold calibration at 300 nodes. The selected threshold is 0.50, where packet delivery ratio reaches its maximum value and energy consumption remains relatively low; the minimum plotted energy consumption occurs at 0.60.

**Figure 12:**
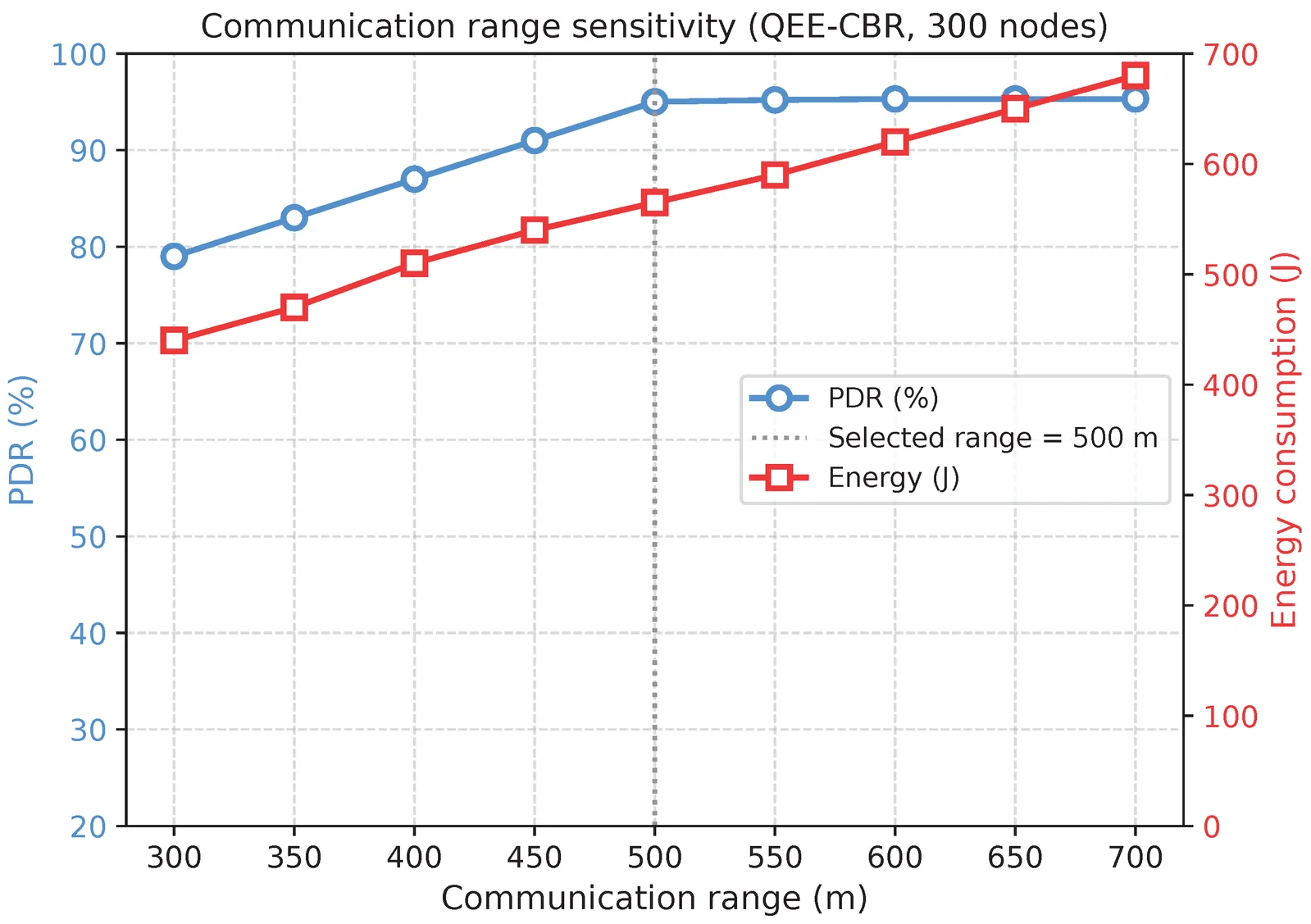
Communication-range sensitivity of QEE-CBR at 300 nodes. The selected range is 500 m, where the packet delivery ratio has effectively plateaued, and the energy consumption remains moderate.

In the one-factor sensitivity experiments performed in Figure 10, Figure 11 and Figure 12, the number of nodes is set to 300 for an intermediate density. The value lies at the midpoint of the evaluated 100–500-node range and avoids selecting either endpoint, where the network is respectively least and most traffic-intensive. Using the same intermediate density for the weight, QoS-threshold, and communication-range experiments also keeps the sensitivity analysis controlled: the node population is fixed, while only the parameter under investigation is varied. The 300-node setting is therefore a reproducible reference condition for isolating parameter effects rather than a claim of an empirically optimal network size.

#### 6.8.1. MCDM Weight Sensitivity

The nominal MCDM weights are α=0.25, β=0.35, ε=0.20, and δ=0.20, which sum to one. A one-factor perturbation is applied to each weight in turn, while the remaining three weights are held at their nominal values. The resulting packet delivery ratio and network lifetime are summarised in Table 10. The residual-energy weight β is also swept more finely in Figure 10, where both metrics improve as β increases from 0.10 to 0.35 and decline as β is increased further to 0.45. The same qualitative behaviour is observed for the other three criteria: peak values occur near the nominal operating point, and performance degrades as any single weight is moved far away from its nominal value.

The shape of the β curve is instructive. When β<0.35, the residual-energy weight is small compared to the other three criteria; thus, the cluster heads elected may not necessarily be energy-rich, and the packet delivery ratio and lifetime will also be reduced. For values of β>0.35, the residual-energy criterion gains more importance than the MCDM score, and the connectivity and spatial terms are acceptable and become marginal, resulting in lower packet delivery ratio and lifetime. The common optimum at β=0.35 is then a balance between energy and topology.

#### 6.8.2. QoS Threshold Calibration

The range of QoS threshold was considered from 0.30 to 0.70. We note, from Figure 11, that when the threshold value is 0.50, the packet delivery ratio is maximal, at about 95%. At this point, the energy consumption is low, and the minimum in the plot is at the threshold value of 0.60, so the chosen value of 0.50 gives more importance to the best observed packet delivery ratio and also has relatively low energy consumption. A lower threshold will have more forwarding candidates and, thus, will be more likely to increase the number of forwarding opportunities but could also result in more unnecessary transmissions and energy usage. The higher the threshold, the more it becomes discriminating and may reject otherwise viable candidates, thus lowering delivery reliability.

Figure 11 shows that there is a trade-off associated with the threshold-based forwarding eligibility. As the threshold decreases (0.30–0.40), more nodes meet the fitness criterion, which may lead to a bigger forwarding opportunity but also to more contention, retransmissions, and energy consumption. When the threshold (0.60–0.70) is higher, there are fewer usable nodes and the the packet delivery ratio is lower. The best packet delivery ratio for the reported sweep is at 0.50, and the lowest energy consumption is observed at 0.60. A compromise of 0.50 is thus a reliability-oriented choice. Future work could involve a joint multi-objective calibration that optimises packet delivery ratio and energy simultaneously.

#### 6.8.3. Communication Range Sensitivity

We varied the communication range from 300 m to 700 m. The communication-range results in Figure 12 show that packet delivery ratio rises rapidly up to 500 m and then exhibits diminishing improvement, whereas energy consumption continues to increase as transmission distance and the number of reachable candidates grow. The selected range of 500 m provides the best observed compromise between connectivity and energy expenditure within the tested range.

Figure 12 shows the well-known relationship between coverage and energy of underwater acoustic networks. A setting with a higher transmission range results in more connected networks, fewer isolated nodes, higher required transmission power, and more neighbours that are in range; this leads to higher per-packet energy usage and more medium access control contention. The near-plateau in the packet delivery ratio above 500 m suggests that no significant gain can be achieved in packet delivery ratio by increasing the range further, while the monotonic increase in energy consumption demonstrates that a range greater than 500 m is not worth the tested configuration.

#### 6.8.4. Node-Speed Sensitivity

The node speed was set by random walk from 0 m/s (static) to 5 m/s, with nominal values of the other parameters. Table 11 presents the resulting packet delivery ratio and lifetime at 300 nodes. The packet delivery ratio is above 94%, and it falls slightly at 5 m/s. At higher speeds, route breakage and extra retransmissions result in higher energy consumption. The proposed hierarchical design is therefore sound in the tested speed range, and higher speeds would require adaptable re-clustering or predictive localisation.

#### 6.8.5. Deployment-Depth Sensitivity

With the radius at 500 m and four levels, the height of the cylinder was changed from 500 m to 1500 m. Table 12 presents the resulting packet delivery ratio and lifetime at 300 nodes. The deeper the deployment, the farther the average distance from the source to the sink, and the more forwarding stages that the packet must pass through; the more the packet’s lifetime decreases, the smaller the packet delivery ratio becomes. The proposed hierarchy still maintains its relative gain with respect to the flat baselines under the tested depths.

#### 6.8.6. Acoustic-Channel Sensitivity

We varied the spreading factor Ω∈{1,1.5,2} and the ambient noise level by ±5 dB while keeping the remaining parameters fixed. Table 13 gives the resulting packet delivery ratio and lifetime at 300 nodes. The packet delivery ratio decreases with Ω and noise, but the relative ordering of the protocols is preserved; that is, the proposed QEE-CBR continues to deliver the highest packet delivery ratio and the longest lifetime under the tested acoustic conditions.

### 6.9. Ablation Study

**Figure 13:**
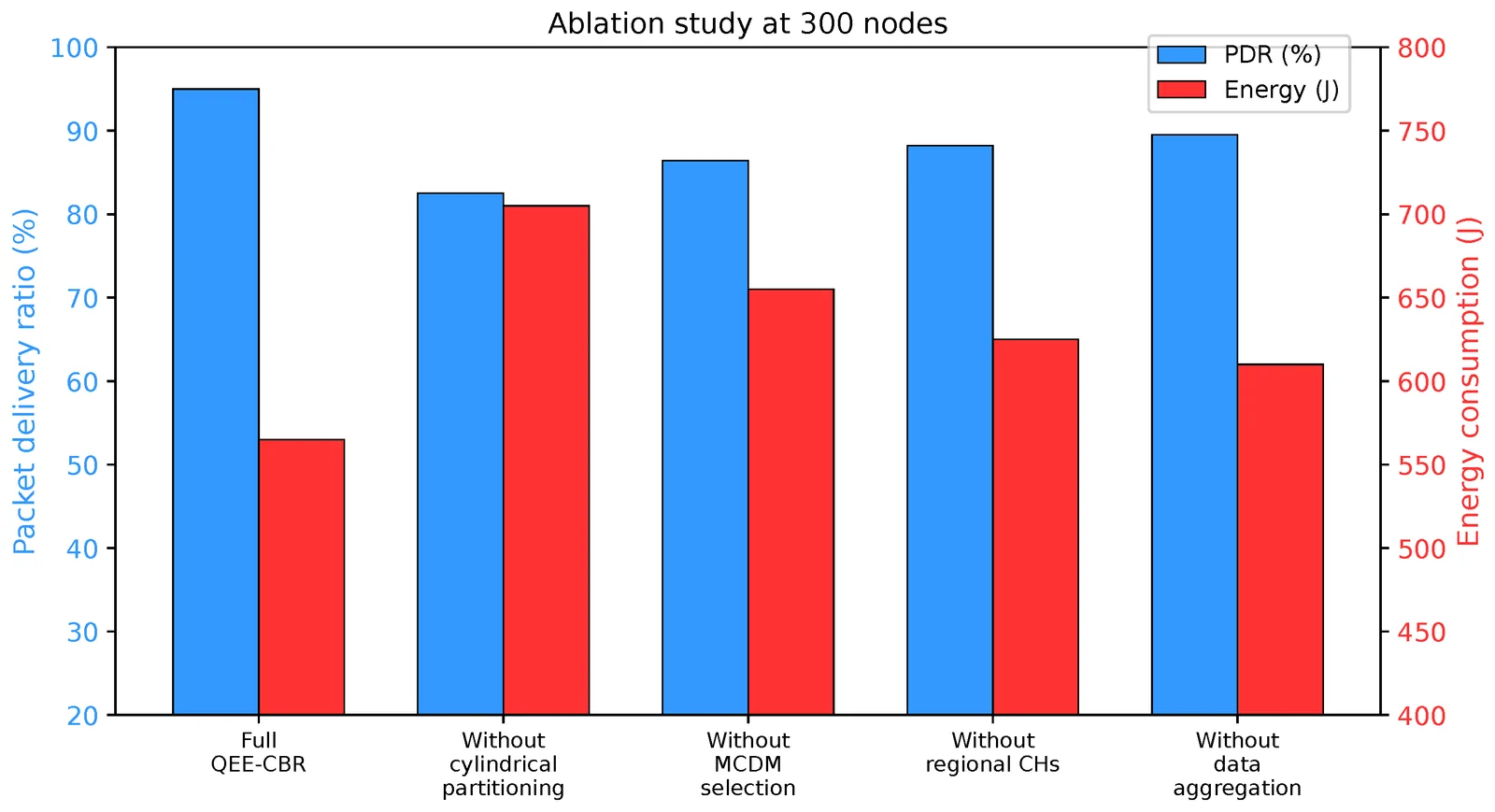
Ablation study at 300 nodes based on the simulation results.

The ablation experiment at 300 nodes included the removal of one of the QEE-CBR components, while preserving the rest of the protocol mechanisms. Figure 13 and Table 14 present the ablation results for the complete protocol and four ablation configurations. This experiment separates the effect of the cylindrical partitioning, MCDM selection of the cluster head, regional cluster heads, and data aggregation mechanisms.

The removal of cylindrical partitioning produces the largest degradation in both metrics: the packet delivery ratio decreases from 95.0% to 82.5%, whereas the energy consumption increases from 565 J to 705 J. Removing MCDM selection reduces the packet delivery ratio to 86.4% and increases energy to 655 J. Removing regional cluster heads gives an 88.2% packet delivery ratio and 625 J, whereas removing data aggregation gives 89.5% and 610 J. These results indicate that the components do not function as independent mechanisms but instead complement each other.

Removing cylindrical partitioning has a significant impact, which is consistent with its purpose of restricting the formation of local clusters and spatially organising forwarding paths. Multi-criteria cluster head selection occurs within the MCDM component, and the regional cluster heads and the aggregation reduce the number of unnecessary long-distance forwardings of unstructured traffic and repeated traffic. The ablation also presents a more positive picture of the integrated design as a whole, but it does not indicate that any individual part is solely responsible for the performance.

### 6.10. Comparison with QE-ORP Protocol

Here, we compare QEE-CBR against our previously proposed QE-ORP protocol [20] across node densities of 100–500. Since the two protocols follow different routing architectures, the comparison is presented as an architectural cross-study reference rather than as part of the four-protocol benchmark suite reported above.

**Figure 14:**
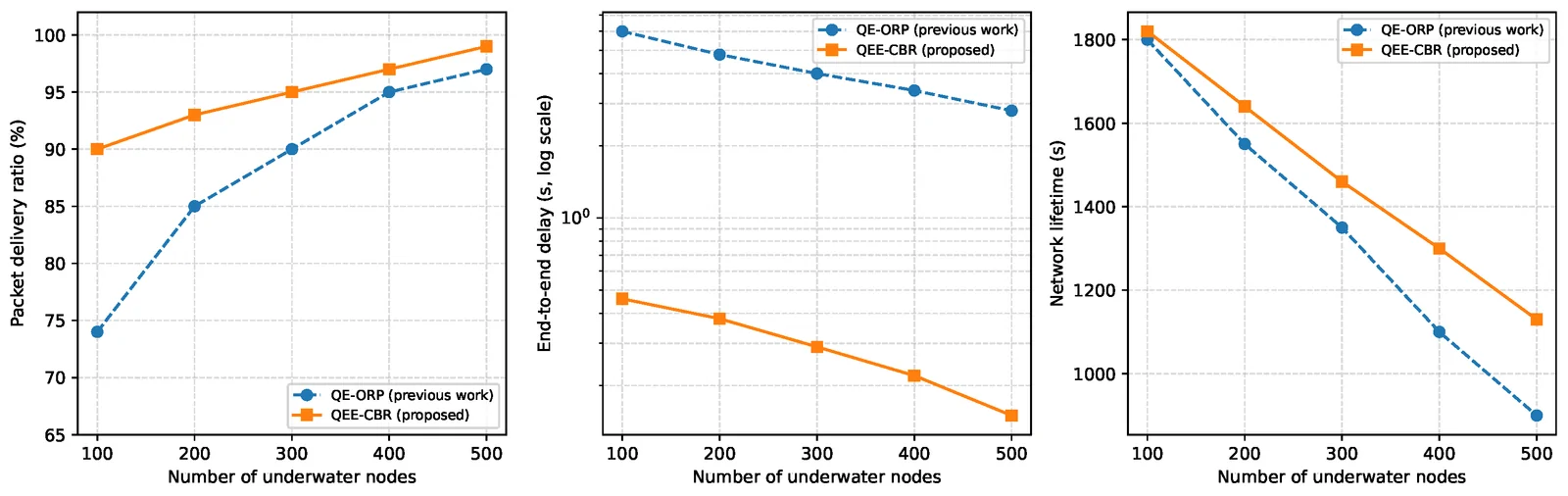
Cross-study comparison of QEE-CBR and QE-ORP for packet delivery ratio, end-to-end delay, and network lifetime.

Figure 14 presents the cross-study comparison with QE-ORP. The panels show packet delivery ratio, end-to-end delay, and network lifetime for the two studies (QE-ORP and QEE-CBR (proposed)). The energy-consumption definitions and evaluation conditions differ between the two studies: the QE-ORP paper defines energy consumption on a per-packet basis, whereas QEE-CBR defines cumulative network energy in joules. The energy panel is therefore omitted from the comparison figure to keep the comparison aligned with metrics that share a comparable basis.

The packet delivery ratio of QEE-CBR is higher and its end-to-end delay is lower than that of QE-ORP at all of the densities cited, and the difference in network lifetime varies from about 20 s at 100 nodes to 230 s at 500 nodes. It is important to note that these differences are described as cross-study observations and do not constitute evidence of superiority because the studies were conducted with different setups. The energy values are not interpreted comparatively, because the two studies’ energy metrics are not based on a common basis.

The packet delivery ratio is 99% and lifetime is 1130 s for QEE-CBR, whereas they are 97% and 900 s for QE-ORP for 500 nodes. Additionally, the routing architectures are different: QE-ORP keeps a flat cooperative forwarding process wherein eligible relays are selected based on the four criteria. In contrast, QEE-CBR has explicit spatial organisation, selects the local cluster heads according to the four criteria, gathers traffic from the members, and forwards the collected traffic through fixed regional cluster heads. QEE-CBR also alters the structure of the network and the route taken by traffic to combine it before it gets to the sink.

#### Comparison with FNCBR Protocol

In this section, we compare the proposed QEE-CBR with the existing FNCBR [40]. The FNCBR protocol is similar to QEE-CBR and is therefore suitable for comparison. FNCBR uses cuboidal partitions, floating surface nodes, and fixed cluster heads at different depths; QEE-CBR uses cylindrical (L,T,S,B) partitioning, locally elected cluster heads, four fixed regional cluster heads distributed across the vertical levels, and a single surface sink. These differences are architectural rather than parametric, and FNCBR cannot be mapped into the four-protocol primary curve set without replacing its cuboidal partitioning and floating-node forwarding with cylindrical equivalents, which would change its core design. Table 15 summarises the architectural differences, and the broader comparison is reflected in the last row of Table 1. The comparison is presented as an architectural cross-study reference. The contribution lies in the integration of explicit spatial organisation, MCDM selection, hierarchical forwarding, and aggregation, rather than in a claim of universal dominance over all routing paradigms.

### 6.11. Overall Interpretation of the Results

The results show that, at the highest evaluated density, QEE-CBR exhibits an unambiguous reliability benefit with no associated delay or cumulative energy. When compared with MLCEE at 500 nodes, the 99% packet delivery ratio of QEE-CBR is approximately 7.6% higher, the delay is approximately 0.15 s, the lifetime is approximately 13% longer at 1130 s, and the energy consumption is approximately 5.2% lower at 730 J. The energy saving is approximately 11% compared with BEEC.

The throughput of QEE-CBR is 425 kbps at 500 nodes, which is 420 kbps for RQAR. These values are not interpretations of the physical capacity of the acoustic link; rather, they are values of the application-data metric defined in Section 5.5. It is therefore much more important to have QEE-CBR with a high packet delivery ratio, lower delay value, controlled energy expenditure, and longer lifetime at the same evaluated density, instead of achieving a higher aggregate throughput.

All of the experiments demonstrate that the performance of QEE-CBR is a function of the interaction between the architectural components. The density sweep provides an overview of the reliability, throughput, delay, lifetime, and energy behaviour; the sensitivity experiments narrow down the operating region of the residual-energy weight, the QoS threshold, the communication range, the node speed, the deployment depth, and the parameters of the acoustic channel; the hop-count/connectivity analysis provides an explanation of the trend of the delays; the localisation experiment investigates the sensitivity to position uncertainty; and the ablation study demonstrates the contributions of the main architectural elements.

In particular, a few observations are relevant for the interpretation of the proposed method. First, the 99% packet delivery ratio for 500 nodes is the highest application-data rate, and the lowest delay value, among the protocols shown. Second, the lifetime of the protocol is 1130 s, and the total energy consumption is 730 J, suggesting that the protocol has the reliability trend and controls energy expenditure. Third, the ablation results indicate that the spatial organisation and the MCDM selection are key components of the protocol because they result in a more significant degradation when they are removed individually when compared to the other two individual components. Last but not least, the QE-ORP comparison shows that the main difference with the previous work is architectural: the proposed approach differs from the previous one in terms of how the network is organised and how traffic is aggregated and forwarded.

The observations are valid for the above-mentioned NS-3 configuration, for the range of density from 100 to 500 nodes, and for the given acoustic parameters. They are found to be effective for the evaluated deployment and give credibility to the evaluation for other deployments in the future.

### 6.12. Application Context

QEE-CBR aims at underwater monitoring with a distributed network of sensing nodes reporting measurements to a surface sink, via a multi-hop acoustic network, that is powered by batteries. A concrete target application is reservoir or aquaculture water-quality monitoring, where sensor nodes are deployed at various locations within the water volume to monitor temperature, dissolved oxygen concentration, pH, and turbidity. Spatial sampling for a reservoir/aquaculture site usually requires a sensing spacing of about 50–100 m to capture the spatial gradient of water quality. This requirement can be mapped onto the deployment volume (R = 500 m, H = 1000 m) that was used for our cylindrical deployment and gives a reasonable number of nodes to be placed in the region (100–500 sensors, the same as the deployment density in our study). The single surface sink is set at the axis of the cylinder (fixed monitoring buoy or shore-connected surface station). The communication constraints of other applications are also suitable for this work, such as oceanographic observation and underwater environmental sensing [62,63,64].

This scenario is a direct mapping of the network model of QEE-CBR. Local cluster heads gather the data from surrounding cluster heads, the regional cluster heads forward the data to the sink, and the (L,T,S,B) partitioning organises the three-dimensional sensing region. The MCDM-based cluster head selection evaluates the candidate nodes in terms of energy and topology [65]. Data aggregation also minimises redundant transmissions in the hierarchy [32,56,66]. The continuity requirement, latency requirement, and autonomy requirement of these applications are translated into the delivery ratio, delay, and throughput requirements of the protocol, respectively, and into the energy and lifetime requirements of the protocol [11,36].

The actual parameters are dependent on the monitoring area, the resolution of the sensing, data rate, characteristics of the modems, and requirements for the lifetime. The number of nodes in the experiments for sensitivity and ablation analysis was set to 300, with the aim of identifying individual routing parameters and architectural components without varying the network density.

## 7. Discussion, Limitations, and Future Work

### 7.1. Discussion

Three architectural features of QEE-CBR are highlighted in the evaluation, namely, (1) (L,T,S,B) cylindrical partitioning as a spatial basis for forming local clusters; (2) residual energy and node degree, centroid proximity, and distance from the block centre are all used in cluster head selection based on MCDM; and (3) fixed regional cluster heads for organising upward forwarding with in-network aggregation. The ablation data can also be used as further evidence of the effect of these components on the performance gained with the tested configuration. The results are still simulation-based, and the interpretation of the throughput, delay, and energy results is limited by the definitions of these metrics and the configuration details presented in this paper.

The delay trend must be considered with the hop-count and connectivity diagnostics. The delay decreases with node density, although the mean hop count increases slightly with a higher density. This trend might be related to better availability of neighbours and better local forwarding, but the results available do not separate the causal contribution of each factor.

According to the sensitivity experiments, the optimal value of the operating point within the range of experimental values is β=0.35, when the value of the QoS threshold is 0.50, and the communication range is 500 m. The weight experiment is interpreted as follows because the other weights were not rebalanced when the change to β was made: these observations describe only the values plotted in the range tested and should not be considered statistically determined optimal values unless there is evidence of independent-run variability.

The cross-study comparison with QE-ORP must be interpreted conservatively. The values show a higher packet delivery ratio and longer lifetime for QEE-CBR; the configurations differ, so the differences are described as cross-study observations. No direct energy-efficiency claim is made from the non-equivalent energy metrics. The contribution is the integration of spatial organisation, multi-criteria cluster head selection, hierarchical regional forwarding, and aggregation.

### 7.2. Limitations

The scope of the present study is defined by the validation methodology, the acoustic and mobility models used for the evaluation, and the parameter ranges considered. The following points clarify the boundaries of the findings and identify directions for extension:

- The results are obtained from NS-3 simulations rather than from a physical acoustic testbed, so they describe protocol behaviour under the stated simulation conditions.
- Node mobility follows a random-walk model at 2–3 m/s, with a supporting node-speed sensitivity analysis at 0–5 m/s. Other patterns, such as current-driven drift or glider motion, are left for future work.
- The acoustic model accounts for frequency-dependent Thorp absorption and spreading, with a Wenz ambient-noise model and an SNR detection threshold. Site-specific effects such as multipath, Doppler spread, surface and bottom reflections, and time-varying ambient noise can be incorporated in future channel representations.
- The localisation-robustness experiment uses additive Gaussian error with σ=10 m. Correlated and non-Gaussian errors, as well as sound-speed-dependent effects, would provide a more detailed picture of positioning uncertainty.
- The sensitivity experiments cover the four MCDM weights, the QoS threshold, the communication range, the node speed, the deployment depth, and selected acoustic-channel parameters. Other parameters, including traffic generation rate and cluster head rotation threshold, can be examined in future studies.
- Table 7 lists the numerical values for the electrical energy-model coefficients, the amplifier energy coefficient, the ambient noise model, the SNR threshold, and the random seed, and the NS-3 simulation configuration is available from the corresponding authors upon reasonable request.
- The four benchmark protocols were evaluated under the stated common simulation conditions while retaining their protocol-specific routing mechanisms. The available materials do not provide complete protocol-specific configuration files or sufficient run-level records to independently reproduce every benchmark curve; this is a limitation of the present evaluation.
- The 100–500-node sweep is a controlled scalability range chosen to reflect large-scale underwater sensing. Extending the evaluation to field-scale density and geometry is a separate, application-specific task.
- The throughput metric is defined at the application-data level and reflects an aggregation-oriented source-generation model (per occupied block). It is therefore not directly comparable to per-node source traffic.

### 7.3. Future Work

As part of our future work, we will extend the evaluation in multiple ways. The protocol will first be tested with a small-scale underwater acoustic modem testbed to validate the protocol under certain physical channel conditions. Second, more realistic mobility and acoustic-channel models will be introduced, such as current-driven drift, multipath, Doppler effects, and non-stationary environmental noise. Third, sensitivity experiments will be repeated with other parameters affecting the load on the network, such as the application generation rate and the threshold for the rotation of cluster heads.

Further work will also investigate correlated localisation errors and adaptive localisation filtering, dynamic regional cluster head placement, congestion-aware forwarding, duty cycling, and cooperative relay mechanisms. Lightweight learning methods, including Q-learning and Multi-Agent Reinforcement Learning (MARL), will be investigated as future adaptive extensions for cluster head selection and route optimisation. Lastly, environmental parameters like temperature, salinity, pressure, and sound-speed profiles will be considered in order to further enhance the physical accuracy of future evaluation tests.

## 8. Conclusions

In this study, we propose the QEE-CBR protocol to solve the problem of reliable and energy-efficient routing in 3D deployments of IoUT. The protocol involves cylindrical (L,T,S,B) partitioning, MCDM-based cluster head selection based on residual energy, node degree, centroid proximity and distance, hierarchical forwarding through fixed regional cluster heads, and in-network data aggregation.

The network lifetime is taken as the time when the first sensor node consumes all of its energy, and the energy consumption of the network is equal to the total energy consumed by all of the alive sensor nodes in the simulation period of 2500 s. These values provide a summary of the configuration evaluated, and the results of the throughput, delay, and energy measures should be read in light of their definition and accounting scope, as outlined in Section 3 and Section 5.5. The experiments performed with localisation-noise, sensitivity, and ablation involve the use of their respective configurations.

The sensitivity experiments obtain a joint operating point of β=0.35 and a communication range of 500 m, and the QoS-threshold sweep reveals that 0.50 maximises the packet delivery ratio, with the minimum energy consumption plotted at 0.60. Further sensitivity tests of node speed, deployment depth, and acoustic channel parameters show that the relative benefit of QEE-CBR over the four primary benchmark protocols remains for the range of test parameters. The ablation study reveals that the removal of any of the cylindrical partitioning, MCDM selection, regional cluster heads, or aggregation deteriorates at least one of the metrics. The difference in architecture between our previous work (QEE-CBR) and the QE-ORP cross-study comparison is the clustered hierarchical forwarding and aggregation that are explicitly represented. The packet delivery ratio and lifetime values are descriptive, and the energy data are used as context-specific because the definitions of the metrics vary.

Furthermore, the distance-dependent amplification should be parameterised with a specific modem model, independent-run statistics should be reported, and the protocol should be tested in a real acoustic testbed and under more channel and mobility conditions. These would increase reproducibility and give further insight into the relevance of observed simulation trends for actual underwater deployments.

## Figures and Tables

**Table 1:** Comparison of existing routing protocols with the proposed QEE-CBR protocol.

Protocol	Clustering Approach	Cluster Head Selection Criteria	Routing Strategy	QoS	Energy	Void Handling	Hierarchical	3D Geometry	Scalability
BEEC [12]	Circular sub-regions	Energy only	Multi-hop with mobile sinks	×	✓	×	×	Circular	Low
MLCEE [13]	Multi-layer clusters	Energy and Bayesian probability	Hierarchical	×	✓	×	✓	Layers	High
RQAR [14]	Flat	TOPSIS: link quality, hop count, congestion, energy	Opportunistic	✓	✓	✓	×	None	Average
EAVARP [15]	Flat	Energy and transmission metrics	Directional	Partial	✓	✓	×	Concentric	Moderate
QERP [38]	Evolutionary clustering	Evolutionary optimisation	Multi-hop	✓	Partial	×	×	None	Moderate
ANCRP [17]	Cubic clusters	Energy only	Anchor-based	Partial	✓	✓	✓	Cuboidal	High
EMLC [16]	Cylindrical multi-level	Energy only	Vertical bottom-up	×	✓	×	✓	Cylindrical	Average
QoS-VAR [39]	Flat	Depth, distance, holding time, energy	Two-hop	✓	✓	✓	×	None	Low
FNCBR [40]	Cuboid clusters	Fixed cluster heads and floating nodes	Hierarchical CH–FN forwarding	×	✓	×	✓	Cuboidal	High
QE-ORP [20]	Flat	Depth, residual energy, link state	Cooperative opportunistic	✓	✓	✓	×	None	Moderate
**QEE-CBR** **(proposed)**	**Cylindrical (** ***L*** **,** ***T*** **,** ***S*** **,** ***B*** **)**	**MCDM: RE, ND, CP, CD**	**Hierarchical with regional cluster heads**	✓	✓	✓	✓	**Cylindrical**	**High**

Abbreviations: CH: cluster head; TOPSIS: Technique for Order Preference by Similarity to an Ideal Solution; RE: residual energy; ND: node degree; CP: centroid proximity; CD: centroid distance; (L,T,S,B): level–track–sector–block; FN: floating node. Legend: ✓ = feature supported; × = feature not supported; Partial = limited or conditional support.

**Table 2:** Broadcast message format for sink.

**Sink node** **identifier**	**Message type** **(information request message)**	**Destination** **address**	**Packet** **sequence number**	**Node** **coordinates**

**Table 3:** Broadcast message format for cluster heads.

**Message type** **(acknowledgment message)**	**Packet** **sequence number**	**Cluster head** **identifier**	**Block** **identifier**	**Destination** **address**	**Coordinates** **(x, y, z)**	**Timestamp**	**Residual** **energy**

**Table 4:** Acknowledgment message from sensor nodes.

**Message type** **(acknowledgment message)**	**Packet** **sequence number**	**Sensor node** **identifier**	**Cluster head** **identifier**	**Block** **identifier**	**Coordinates** **(x,y,z)**	**Residual** **energy**	**Time** **stamp**

**Table 5:** Information request message for joining the cluster.

**Message type** **(information request message)**	**Sensor node** **identifier**	**Cluster head** **identifier**	**Block** **identifier**	**Residual** **energy**	**Node** **coordinates**

**Table 6:** Data packet format.

**Regional cluster head** **hop identifier**	**Next regional cluster head** **hop identifier**	**Layer** **number**

**Table 7:** Hardware/software execution environment and simulation parameters used for the evaluation.

Parameter	Value
**Execution environment**	
Simulator	NS-3.40
Host processor	Apple M1
Host memory	16 GB RAM
Host storage	512 GB SSD
Operating system	macOS Monterey
Compiler	GNU g++ 11.2.0
**Topology and traffic**	
Bounding box	1000×1000×1000 m^3^
Sensor node population	100, 200, 300, 400, 500
Vertical levels	4 (250 m each)
Radial tracks	1
Sectors per level	8 ($45^{\circ }$ each)
Radial blocks per sector	4 (125 m each)
Regional cluster heads	4 fixed, one per level
Surface sink	1 fixed at the cylinder axis
Data packet size	200 bytes
Control packet size	5 bytes
**Acoustic and propagation model**	
Acoustic frequency	10 kHz
Acoustic bandwidth	4 kHz
Propagation speed	1500 m/s
Propagation-loss model	Thorp; Ω=1.5, $d_{0}=1$ m
Ambient noise model	Wenz shipping + wind + thermal
SNR detection threshold, $\gamma_{0}$	10 dB
Communication range	500 m
Cluster-joining distance threshold	400 m
**Energy and routing configuration**	
Initial sensor node energy	5 J per node
Mobility model	Random walk; 2–3 m/s
MCDM weights (α,β,ε,δ)	(0.25,0.35,0.20,0.20)
QoS forwarding threshold	0.50
Simulation runtime	2500 s
**Electrical energy-model parameters**	
Electronics coefficient, $E_{elec}$	50 nJ/bit
Amplifier energy coefficient, $E_{amp}$	0.01 nJ/bit/m^1.5^
Receiver power term, $P_{rx}$	10 mW
Data-aggregation coefficient, $E_{DS}$	5 nJ/bit

**Table 8:** Comparison of average hop count across different node densities.

Protocol	100	200	300	400	500
BEEC	4.5	5.0	5.5	6.0	6.2
MLCEE	4.6	5.0	5.3	5.7	6.0
RQAR	5.0	5.5	6.0	6.5	7.0
EAVARP	4.8	5.2	5.6	6.1	6.5
**QEE-CBR (proposed)**	**3.5**	**3.8**	**4.1**	**4.3**	**4.6**

**Table 9:** Impact of 10 m Gaussian localisation noise on network performance.

Protocol	Packet Delivery Ratio (%)	Delay (s)	Energy (J)	Throughput (kbps)
BEEC	73.4 (↓7.1%)	0.35 (↑9.4%)	884 (↑7.8%)	325 (↓7.1%)
MLCEE	86.6 (↓5.9%)	0.19 (↑5.6%)	813 (↑5.6%)	359 (↓5.5%)
RQAR	78.7 (↓8.5%)	0.23 (↑9.5%)	871 (↑8.9%)	386 (↓8.1%)
EAVARP	83.9 (↓6.8%)	0.23 (↑9.5%)	835 (↑7.1%)	365 (↓6.4%)
**QEE-CBR (proposed)**	**96.0 (↓3.0%)**	**0.16 (↑6.7%)**	**753 (↑3.2%)**	**413 (↓2.8%)**

**Table 10:** One-factor MCDM weight sensitivity at 300 nodes.

Perturbed Weight	Value	Packet Delivery Ratio (%)	Lifetime (s)
Nominal (α,β,ε,δ)	(0.25,0.35,0.20,0.20)	95.0	1460
α (node degree)	0.10	91.5	1370
α (node degree)	0.40	92.0	1400
β (residual energy)	0.10	88.5	1310
β (residual energy)	0.45	91.0	1390
ε (centroid proximity)	0.05	92.5	1405
ε (centroid proximity)	0.40	91.8	1400
δ (block-centre distance)	0.05	92.0	1395
δ (block-centre distance)	0.40	91.5	1390

**Table 11:** Node-speed sensitivity of QEE-CBR at 300 nodes.

Node Speed (m/s)	Packet Delivery Ratio (%)	Lifetime (s)	Energy (J)
0 (static)	96.2	1505	545
1	95.8	1490	552
2	95.0	1460	565
3	94.3	1430	578
5	91.6	1370	602

**Table 12:** Deployment-depth sensitivity of QEE-CBR at 300 nodes, with four vertical levels.

Deployment Height (m)	Packet Delivery Ratio (%)	Lifetime (s)	Energy (J)
500	96.4	1510	548
1000 (nominal)	95.0	1460	565
1500	92.7	1385	590

**Table 13:** Acoustic-channel sensitivity of QEE-CBR at 300 nodes.

Configuration	Packet Delivery Ratio (%)	Lifetime (s)	Energy (J)
Ω=1.0, nominal noise	96.8	1495	552
Ω=1.5, nominal noise (baseline)	95.0	1460	565
Ω=2.0, nominal noise	91.5	1400	588
Ω=1.5, −5 dB noise	95.7	1470	560
Ω=1.5, +5 dB noise	93.0	1420	578

**Table 14:** Ablation study at 300 nodes based on the simulation results.

Configuration	Packet Delivery Ratio (%)	Energy (J)
Full QEE-CBR (proposed)	95.0	565
Without cylindrical partitioning	82.5	705
Without MCDM selection	86.4	655
Without regional cluster heads	88.2	625
Without data aggregation	89.5	610

**Table 15:** Architectural comparison of FNCBR and QEE-CBR.

Feature	FNCBR	QEE-CBR (Proposed)
Deployment geometry	Cuboidal sub-regions	Cylindrical right cylinder
Spatial identifier	Region index	(L,T,S,B)
Surface support	Floating surface nodes	Single fixed surface sink
Cluster head type	Fixed cluster heads at different depths	Locally elected MCDM cluster heads
Cluster head selection criteria	Fixed assignment	RE, ND, CP, CD (Equation (22))
Forwarding path	Hierarchical CH–FN	Hierarchical CH–RCH–sink
Data aggregation	Per cluster head	Per cluster head and per regional cluster head
Energy sources	Battery-powered nodes and floating nodes	Battery-powered nodes, externally powered regional cluster heads

## Data Availability

All supported data of this study are available from the first author upon reasonable request.

## Nomenclature

The following abbreviations and mathematical symbols are used in this manuscript.

Abbreviations	
AI	Artificial Intelligence
ANCRP	Anchor-Nodes-Assisted Cluster-Based Routing Protocol
BEEC	Balanced Energy-Efficient Clustering
BP	Beacon Packet
CD	Centroid Distance
CH	Cluster Head
CNPS	Cylindrical Network Partitioning Scheme
CP	Centroid Proximity
DT	Distance Threshold
E2E	End-to-End
EAVARP	Energy-Aware Void Avoidance Routing Protocol
EMLC	Energy-Aware Multi-Level Clustering Scheme
FNCBR	Floating-Nodes-Assisted Cluster-Based Routing
GNU	GNU’s Not Unix
IoUT	Internet of Underwater Things
(*L*,*T*,*S*,*B*)	Level–Track–Sector–Block
MARL	Multi-Agent Reinforcement Learning
MCDM	Multi-Criteria Decision-Making
MLCEE	Multi-Level Clustering Energy-Efficient Routing
ND	Node Degree
NN	Neighbour Node Set
NS-3	Network Simulator 3
NTP	Network Throughput
ODFS	Opportunistic Directional Forwarding Strategy
PDR	Packet Delivery Ratio
QE-ORP	QoS-Aware and Energy-Optimised Cooperative Opportunistic Routing Protocol
QEE-CBR	Quality-of-Service-Aware and Energy-Efficient Cluster-Based Routing
QERP	QoS-Aware Evolutionary Routing Protocol
QoS	Quality of Service
QoS-VAR	QoS-Based Void Avoidance Routing Technique
RAM	Random-Access Memory
RCH	Regional Cluster Head
RE	Residual Energy
RQAR	Reliable QoS-Aware Routing
SDN	Software-Defined Networking
SK	Sink Node
SN	Sensor Node
SNR	Signal-to-Noise Ratio
SSD	Solid-State Drive
TDMA	Time Division Multiple Access
ToA	Time of Arrival
TOPSIS	Technique for Order Preference by Similarity to an Ideal Solution
Mathematical symbols	
$A_{i}$	Set of transmission, reception, and aggregation operations performed by node *i*
*B*	Number of radial blocks per sector
$B_{\mathrm{recv}}$	Application-payload bytes represented by successfully received sink data
$CC_{i}$	Centroid proximity score for candidate node *i*
$CC_{C}$	Cylindrical centroid representation of cluster C
C	Set of nodes forming a cluster
CR(R,H)	Cylindrical region with radius *R* and height *H*
$D_{i}$	Distance of candidate node *i* from the block centre (m)
$D_{\min},D_{\max}$	Minimum and maximum values of $D_{i}$ over the candidate nodes in a cluster
$D_{i}^{norm}$	Min–max normalised value of $D_{i}$ over the candidate nodes in a cluster
d(i,j)	Euclidean distance between nodes *i* and *j* (m)
$d_{0}$	Reference distance used in the propagation and amplifier-power models (m)
$d_{\mathrm{km}}$	Link distance expressed in kilometres (km)
*d*	Transmission distance between nodes (m)
$d_{c,i}$	Distance from candidate node *i* to its cluster centroid (m)
$d_{c,\min},d_{c,\max}$	Minimum and maximum values of $d_{c,i}$ over the candidate nodes in a cluster
$D_{\mathrm{avg}}$	Average end-to-end delay (s)
DT	Distance threshold for cluster joining (m)
*E*	Energy consumption of a node (J)
$E_{a}$	Energy consumed by a single transmission, reception, or aggregation operation *a* (J)
$E_{DA}\left(l\right)$	Energy consumed during data aggregation of an *l*-bit packet (J)
$E_{\mathrm{amp}}$	Amplifier energy per bit at the reference distance (J/bit)
$E_{\mathrm{DS}}$	Energy dissipated per bit during data aggregation processing (J/bit)
$E_{\mathrm{elec}}$	Energy dissipated per bit by transmitter and receiver electronics (J/bit)
$E_{I}$	Initial energy of a sensor node (J)
$E_{\mathrm{total}}$	Total energy consumption of all sensor nodes (J)
$E_{tx}\left(l,d\right)$	Energy required to transmit an *l*-bit packet over distance *d* (J)
$E_{rx}\left(l\right)$	Energy required to receive an *l*-bit packet (J)
$F(V,CH_{i})$	Weighted decision function for selecting cluster head $CH_{i}$
*f*	Acoustic signal frequency (kHz)
*G*	Lumped transmitter/receiver gain factor in the SNR model
*H*	Height of the cylindrical deployment area (m)
*h*	Height (thickness) of each vertical level (m)
$h_{l}$	Height of a single vertical level computed as H/L (m)
*k*	Number of nodes in a cluster
*L*	Number of vertical levels
*l*	Packet length (bits)
L(d,f)	Linear propagation loss factor
*N*	Total number of sensor nodes in the network
N(f)	Ambient noise power spectral density at frequency *f*
$N_{\mathrm{delivered}}$	Number of unique application packets successfully delivered to the sink
$N_{\mathrm{generated}}$	Number of unique application packets generated by source nodes
$ND_{i}$	Node degree of node *i*
$ND_{\min},ND_{\max}$	Minimum and maximum values of $ND_{i}$ over the candidate nodes in a cluster
$ND_{i}^{norm}$	Min–max normalised value of $ND_{i}$ over the candidate nodes in a cluster
$NN_{i}$	Neighbour node set of node *i*
$NZ(R,H,R_{c})$	Network zone with radius *R*, height *H*, and communication range $R_{c}$
$P_{amp}\left(d\right)$	Distance-dependent electrical transmitter–amplifier power term (W)
$P_{rx}$	Receiver power term used in the electrical energy model (W)
$P_{tx}$	Transmit power used in the SNR model (W)
*R*	Radius of the cylindrical deployment area (m)
$R_{b}$	Nominal link data rate (bit/s)
$R_{c}$	Communication or transmission range of a sensor node (m)
$R_{\mathrm{tab}}\left(CH_{i}\right)$	Routing or information table maintained by cluster head $CH_{i}$
$R_{\mathrm{tab}}\left(N_{i}\right)$	Routing or information table maintained by sensor node $N_{i}$
$RE_{i}$	Residual energy of node *i* (J)
$RE_{\min},RE_{\max}$	Minimum and maximum values of $RE_{i}$ over the candidate nodes in a cluster
$RE_{i}^{norm}$	Min–max normalised value of $RE_{i}$ over the candidate nodes in a cluster
*S*	Number of sectors per track
TL(d,f)	Transmission loss relative to a 1 m reference distance (dB)
$T_{tx},T_{rx}$	Modelled transmission and reception durations of a packet (s)
$T_{n}$	Number of radial tracks
$T_{i}^{\mathrm{recv}}$	Delivery time of application packet *i* at the sink (s)
$T_{i}^{\mathrm{send}}$	Source transmission time of application packet *i* (s)
$T_{\mathrm{NL}}$	Network lifetime (s)
$T_{\mathrm{obs}}$	Observation interval used for throughput measurement (s)
$T_{\mathrm{sim}}^{\mathrm{first}/\mathrm{dead}}$	Time until the first sensor node exhausts its energy (s)
α	Weighting coefficient for node degree in MCDM
α(f)	Thorp absorption coefficient at frequency *f* (dB/km)
β	Weighting coefficient for residual energy in MCDM
$\gamma_{0}$	SNR detection threshold (dB)
δ	Weighting coefficient for block-centre distance in MCDM
Δr	Radial width of a single block within a sector (m)
Δθ	Angular width of a single sector (rad)
ε	Weighting coefficient for centroid proximity in MCDM
N	Set of all sensor nodes in the network
I(·)	Indicator function, equal to 1 if the condition is true and 0 otherwise
Ω	Spreading factor (Ω=1 cylindrical, Ω=1.5 practical, Ω=2 spherical)
σ	Standard deviation of Gaussian localisation noise (m)
${\bar{x}}_{C},{\bar{y}}_{C},{\bar{z}}_{C}$	Cartesian coordinates of the cluster centroid of cluster C
${\bar{x}}_{B},{\bar{y}}_{B},{\bar{z}}_{B}$	Cartesian coordinates of the geometric centre of the local block
(r,θ,z)	Cylindrical coordinates: radial distance, azimuthal angle, and vertical distance
$\left(x_{i},y_{i},z_{i}\right)$	Cartesian coordinates of node *i*
